# Trying to Kill a Killer; Impressive Killing of Patient Derived Glioblastoma Cultures Using NK-92 Natural Killer Cells Reveals Both Sensitive and Highly Resistant Glioblastoma Cells

**DOI:** 10.3390/cells14010053

**Published:** 2025-01-05

**Authors:** Jane Yu, Hyeon Joo Kim, Jordyn Reinecke, James Hucklesby, Tennille Read, Akshata Anchan, Catherine E. Angel, Euan Scott Graham

**Affiliations:** 1 Department of Molecular Medicine and Pathology, School of Medical Sciences, Faculty of Medical and Health Sciences, University of Auckland, Auckland 1023, New Zealand; jane.yu@auckland.ac.nz (J.Y.); hyeonjookim98@gmail.com (H.J.K.); jordyn.reinecke@petermac.org (J.R.); tennille.read@auckland.ac.nz (T.R.); a.anchan@auckland.ac.nz (A.A.); 2 Centre for Brain Research, University of Auckland, Auckland 1023, New Zealand; james.hucklesby@auckland.ac.nz; 3 School of Biological Sciences, Faculty of Science, University of Auckland, Auckland 1023, New Zealand; c.angel@auckland.ac.nz

**Keywords:** glioblastoma, natural killer cells, NK-92 cells, immunotherapy, killing assays, brain tumour biology

## Abstract

The overall goal of this work was to assess the ability of Natural Killer cells to kill cultures of patient-derived glioblastoma cells. Herein we report impressive levels of NK-92 mediated killing of various patient-derived glioblastoma cultures observed at ET (effector: target) ratios of 5:1 and 1:1. This enabled direct comparison of the degree of glioblastoma cell loss across a broader range of glioblastoma cultures. Importantly, even at high ET ratios of 5:1, there are always subpopulations of glioblastoma cells that prove very challenging to kill that evade the NK-92 cells. Of value in this study has been the application of ECIS (Electric Cell–Substrate Impedance Sensing) biosensor technology to monitor the glioblastoma cells in real-time, enabling temporal assessment of the NK-92 cells. ECIS has been powerful in revealing that at higher ET ratios, the glioblastoma cells are acutely sensitive to the NK-92 cells, and the observed glioblastoma cell death is supported by the high-content imaging data. Moreover, long-term ECIS experiments reveal that the surviving glioblastoma cells were then able to grow and reseed the culture, which was evident 300–500 h after the addition of the NK-92 cells. This was observed for multiple glioblastoma lines. In addition, our imaging provides evidence that some NK-92 cells appear to be compromised early, which would be consistent with potent evasive mechanisms by the glioblastoma tumour cells. This research strongly highlights the potential for NK-92 cells to kill glioblastoma tumour cells and provides a basis to identify the mechanism utilised by the surviving glioblastoma cells that we now need to target to achieve maximal cytolysis of the resistant glioblastoma cells. It is survival of the highly resistant glioblastoma clones that results in tumour relapse.

## 1. Introduction

Survival statistics for glioblastoma patients are among the worst for all cancers. The median survival, reported globally, has hardly changed in the last 15 years, even including patients involved in clinical trials for various immunotherapies [1,2,3]. Median OS can be 10–15 months and is strongly influenced by the course of treatments [4,5], patient’s age [1,5], metabolic disease status/other comorbidities [1], tumour location [5,6] and whether they are able to receive surgical tumour debulking [1,2,7]. Shockingly, median overall survival (OS) can be less than four months in elderly patients with no options for surgical intervention or treatment [7]. In recent glioblastoma clinical trials, the benefits of various monoclonal antibody checkpoint immunotherapies have shown modest increases in median OS [8] but nowhere near the successes observed in other cancers such as advanced melanoma [9,10,11]. The collective state of play for glioblastoma therapy with a line of sight to a cure is currently poor, and as such we desperately need to find solutions to target and kill the most resistant and resilient tumour cells.

Natural Killer (NK) cells are a promising option, being one of our immune system’s innate repertoires designed to detect unhealthy cells, virally infected cells and some tumour cells. Healthy cells deploy an abundance of human leukocyte antigen (HLA)-class 1 molecules that are inhibitory signals to NK cells. These molecules bind inhibitory receptors on the NK cells, confirming that the cell is healthy and should be left alone [12,13] as it does not pose a threat. However, when cells become unhealthy, they downregulate HLA class 1 molecules (known as missing-self) and upregulate cell-surface activation ligands [14,15,16] and cytokines, which can then override the NK cell’s inhibitory status. These are key signals required by the NK cells to kill the respective target [14,15].

There are several options to consider in terms of NK cell sources. The most obvious are primary NK cells from blood, however these have a range of known limitations especially around cell yields and high levels of inhibitory receptors. Of the various “NK-cell lines” the NK-92 cell line is one of the most promising. The original NK-92 line was isolated by Hans Klingemann and colleagues in 1992. The NK-92 donor was a 50-year-old white male with rapidly progressive and aggressive clonal non-Hodgkin’s lymphoma [17,18]. Since then, considerable research has indicated their potential to kill cancer cells [18,19,20,21] and variants of the NK-92 line exist that have been genetically modified to provide additional functionality or survival benefit to the NK-92 cells [22,23]. See [24] for an extensive review on the history and impact of the NK-92 cell platform. Importantly, the NK-92 cells are also approved by the U.S. Food and Drugs Administration (FDA) for phase I and phase II clinical trials as an adoptive cell therapy and have been administered to more than 500 cancer patients [19,24,25].

The goal of this research was to ascertain the threshold levels of sensitivity of different glioblastoma cultures to NK-mediated killing. We began this research with primary allogeneic blood-derived NK cells and quickly switched to the NK-92 Natural Killer cells for a range of reasons. We hypothesised that in any given culture of glioblastoma (or any other form of cancer), there would exist tumour cells that are sensitive to NK cells and will be killed. Equally there will be tumour cells that are highly resistant, where they are not recognised at all by the NK cells and/or where the NK cells are deliberately suppressed by the glioblastoma cells. Importantly, for the NK-92 work we observed impressive levels of glioblastoma cell loss and killing at ET ratios of 5:1, which in some experiments reached 80–90% glioblastoma cell loss. Importantly we also observed promising glioblastoma killing at the lower 1:1 ratio. The most important observation was that complete glioblastoma cell cytolysis was never achieved. Inferring resistant clones were indeed present. Most curiously, we present evidence that some NK-92 cells were killed within 24 h of co-culture through mechanisms that are currently unknown. In addition, we reveal that those glioblastoma cells that survive the initial killing phase reseed and regrow the cultures. Identification of the molecules involved in glioblastoma’s suppression and evasion of NK-92 is our next goal along with the aim of identifying targets to potentiate killing of resistant glioblastoma cells by NK-92. This is essential to achieve maximal killing of the most resistant glioblastoma tumour-initiating cells.

## 2. Methods Section

### 2.1. Cell Culture Protocol of Glioblastoma Cell Lines

Different patient-derived glioblastoma lines were sourced from the Auckland Cancer Society Research Centre (ACSRC) under collaboration with Dr Graeme Finlay and Professor Bruce Baguley as detailed previously [26,27,28]. These cultures (NZB11, NZN12, NZB13, NZB14, NZB15 and NZB19) were all grown in α-MEM (Gibco Cat#12561056) media supplemented with 5% FBS and insulin-transferrin-sodium-selenite (ITS) (Sigma Cat#11074547001, St. Louis, MO, USA). These media are referred to here as complete glioblastoma media. The NZB lines are relatively adherent and require full media change every 3–5 days with a flask change every 10–14 days. The NZB lines are cultured in a low oxygen incubator set at 37 °C, 5% O_2_ and 5% CO_2_ to mimic the tumour microenvironment of glioblastoma. The glioblastoma cells were harvested with Accutase (Sigma Cat#A6964-500ML). Accutase is considered gentler than trypsin and so can dissociate the cells whilst maintaining their cell-surface phenotype. Accutase was neutralised by the same volume of serum-containing glioblastoma media and the cell suspension centrifuged at 300× *g* for 5 min in a 15 mL Falcon tube. After centrifugation the cell pellet was aspirated, resuspended in 1 mL of glioblastoma media and the cells were counted using Trypan blue and a haemocytometer.

### 2.2. Culture of NK-92 Cells

NK-92^®^ (ATCC CRL-2407) cells were purchased from ATCC and cultured following the advised protocol in an RPMI-based media. Complete NK-92 media contain RPMI 1640 (Gibco, Grand Island, NY, USA; Invitrogen, Carlsbadm, CA, USA) supplemented with 12.5% horse serum (HS) (CAT#16050122), 10% foetal bovine serum (FBS; New Zealand sourced), 2 mM Glutamax (Cat#35050061), 1% Penstrep (CAT#15140148, 100×), 1 mM sodium pyruvate (Cat#11360070), non-essential amino acids (NEAA) (Cat#1114005) and 5 ng/mL IL-2 (Biolegend, San Diego, CA, USA, Cat#589102); NK-92 cells are IL-2 dependent. NK-92 cells were grown under normoxic conditions (37 °C, 21% O_2_ and 5% CO_2_) and received a full media change and cell split change every 2–3 days. NK-92 cells typically grow in suspension, as large round clusters of cells maximising contact with neighbouring cells (see Supplemental Figure S1).

### 2.3. Glioblastoma Killing Assays Using NK-92 Cells

Patient-derived glioblastoma lines were harvested and plated in flat bottom 96-well plates (ThermoFisher Cat#167008, Waltham, MA, USA) at a density of 10,000 cells/well. Cells were plated in 100 µL of complete glioblastoma media and left to adhere for ~48 h in the low oxygen incubator. For the killing assay, the NK-92 cells were harvested and rinsed with RPMI and cells counted using a hemocytometer. NK-92 cells were resuspended in complete NK-92 media at the desired titration. Three different effector: target (E:T) ratios were chosen in order to cover a spectrum of high to low E:T ratio—5:1; 1:1; 1:5. Then, 100 µL of NK-92 cell suspension was added directly on top of adhered glioblastoma cells and placed in the incubator.

### 2.4. ECIS Monitoring of NK-92 Mediated Killing of Glioblastoma Cells

ECIS 96W1E+PET arrays (Applied BioPhysics, Troy, NY, USA) were used to measure glioblastoma cell adhesion as a surrogate of cell health. The plates were pre-coated with 10 mM L-Cysteine to stabilise the electrodes, then washed once with Milli Q water prior to cell seeding. Glioblastoma cells were cultured in complete media and harvested as described previously using Accutase. Cells were seeded at 10,000 glioblastoma cells per well in 100 µL of complete media. Resistance was measured at 4000 Hz and monitored continuously throughout the experiment. Typically, NK-92 cells were added after the glioblastoma cells had been in culture for 48–50 h to enable strong stable adhesion, and this time point is indicated on the respective ECIS graphs. The resistance values indicate the net glioblastoma cell adhesion across the ECIS electrodes. In all ECIS assays, each respective treatment or control is set up using at least 4–6 independent wells on the 96-well plate. In addition, wells that have media only, designated as cell-free wells, show the basal resistance across the electrodes in the absence of cells. This is indicated on each curve as the grey flat line positioned around 1600–1700 ohms.

### 2.5. ECIS Application and Contextualisation

In these assays, the glioblastoma adhesion is used as a surrogate to monitor the glioblastoma cell health over time (in real time and continuously). The value of ECIS technology is the real-time nature of the measurements and for revealing when the glioblastoma cells respond to the NK-92 cells. As NK-92 cells start to induce glioblastoma cell compromise and cell death, glioblastoma adhesion will be lost, and the resistance will decline. This loss in resistance will be sustained when the glioblastoma cells are killed. In contrast, where the loss in resistance is acutely transient (where the loss in resistance normalises within hours), this typically relates to an acute morphological change in the cells (e.g., shrinkage, contraction, migration), rather than cell death. Dead cells do not provide a significant barrier to current flow and have low resistive properties.

### 2.6. Immunocytochemical Stain of NK-92-Glioblastoma Co-Cultures

The key aspect of this assay was clear discrimination of the NK-92 cells and the glioblastoma cells for the high-content image analysis. Several highly expressed NK-markers (CD45, CD28 and CD56) were assessed to see which would specifically identify the NK-92 by immunocytochemistry post-fixation. CD45 proved to be the best and provided the strongest signal post-fixation. NK-92 cells also express CD28 and CD56. However, the antibody we used for CD28 stained less intensely post-fixation and unexpectedly the glioblastoma cells also expressed CD56 (NCAM), rendering CD56 unsuitable to discriminate the NK-92 cells . The glioblastoma cells were completely negative for CD45.

NK-92 cells were added to the wells at desired E:T ratios of 5:1, 1:1 and 1:5. Media control wells were set up at the same time on the same plate. After NK-92 cells were added, plates were returned to the incubator for the duration of the experiments. For ECIS plates, cell impedance was measured at 4000 Hz and monitored continuously for the duration of the experiment, while for the standard 96-well plates, they were removed from the incubator at desired time points for immunocytochemical analysis.

For immunocytochemical analysis of glioblastoma cell loss following NK-92 cell treatment, the cells were fixed in 4% paraformaldehyde (PFA) for 10 min and then washed once with PBS. Cells were permeabilised for a further 10 min in 0.1% PBS-Triton X-100 (PBS-T). Cells were washed 3 times with PBS-T and stored at 4 °C in PBS. For immunocytochemical staining, the PBS-T was removed from the wells, and cells were then blocked in 1% bovine serum albumin (BSA in PBS) for 45 min and washed 3 times for 10 min in 0.1% PBS-T. Primary mouse anti-CD45 antibody (Abcam CAT# ab30470, Cambridge, MA, USA) was diluted in 1% BSA, as per Table 1, and incubated with cells for 1 h at room temperature on a plate rocker. After primary antibody incubation, cells were washed 3 times with PBS-T and then incubated with 1:400 goat anti-mouse secondary antibody, Alexa Fluor™ 594 (Thermo Fisher, CAT#A11005) for another 1 h at room temperature on a rocker. Cells were then washed again 3 times with PBS-T, followed by F-actin staining using ActinGreen™ 488 ReadyProbes™ (Thermo Fisher, CAT# R37110) and counterstained with Hoechst 33342 (Invitrogen CAT# H3570) at a dilution of 1:10,000 in 1% BSA/PBS for 20 min at room temperature; then cells were washed with PBS once and stored in PBS at 4 °C until imaging.

### 2.7. Image Acquisition and Analysis Pipeline

The PerkinElmer Operetta high-content imaging system at the School of Biological Sciences was used to obtain images for subsequent cell loss analysis and quantification of each experiment. The Operetta is a high-throughput microplate imaging system used for high-content analysis and is fully automated with high resolution autofocus and image capture. Each ECIS experiment had three separate 96-well companion plates, corresponding to either 2, 4 or 24 h co-culture time points. Each well was imaged at 9 regions that were consistently positioned across all wells to ensure even and consistent imaging coverage of the wells. This approach was to maximise the accuracy of cell counting across the wells. Each treatment group (glioblastoma-only control, 5:1, 1:1 and 1:5 E:T ratios) was applied across 3–4 replicate wells in each individual experiment. Therefore, each individual treatment produced a total of 27–36 images per plate for quantification.

For the initial setup and optimisation of the Operetta system, one of the replicate wells for the 5:1 treatment group at 2 h was used, as this provided an adequate number of both GBM cells and NK92 cells. The plate type was selected as 96 NUNC Microwell plate F96. Three imaging channels were used, and all were set at auto contrast: Alexa 488 for Actin stain (colour #4AFF00), DAPI for DAPI nuclei staining (colour #0028FF) and Alexa 594 for CD45 on the NK-92 cells (colour #FF8900). Magnification was set as 20× long WD, imaging mode set to fluorescence and excitation set to 50%, with the excitation source being a 300W Xenon lamp. Image control was set at “highlight colouring” to enhance the images, otherwise they appeared too dark when captured. To define the z-plane (stack) and therefore the most ideal distance of the objective lens to the sample, 12 planes were chosen, the first beginning at −8.0 µm and the last at 14.0 µm. The stack definition was then evaluated by taking test images at each plane within the chosen height parameters. The height of ~4.0 µm often displayed the sharpest image for actin and CD45, while 2.0 µm was sharpest for DAPI, as the other heights resulted in diffuse images and therefore were used for imaging of all plates. The Operetta was then used to will capture controlled time-course imaging of each well, taking a total of approximately 26 min for all 32 wells per plate. When the imaging was completed, all images were exported to the Columbus Helper online application.

Cell loss quantification analysis was conducted by using the CellProfiler software application (version 4.2.7) with an established pipeline consisting of several modules used for all image analyses. CellProfiler is a method used to filter out the NK-92 cells on each image based on positive CD45 expression, resulting in final counts of only the number of glioblastoma cells. A file of all images taken on the Operetta is opened in CellProfiler, and the established pipeline set is analysed on all images sequentially. Firstly, the “Identify Primary Objects” module is used for nuclei identification of both GBM and NK92 cells and is set as DAPI for the input image at an object diameter of 10–100-pixel units. Any objects that are touching the border of the image are included. The “Dilate Objects” module is then used to expand the boundaries of the objects by 5 pixel units, as the CD45 expression is on the outer membrane of the NK-92 cells. “Measure Object Size Shape” and “Measure Object Intensity” modules are used to measure several different features of the objects, the most important being the intensity of CD45. The “Display Histogram” module produces a histogram of the mean intensity of CD45 for all objects, where a large peak generally below 0.003 pixels corresponds to the glioblastoma cells. The “Filter Objects” module is used to filter out objects with a CD45 mean intensity above 0.003 pixels, to give final filtered object values corresponding only to the glioblastoma cells. Once complete, all data are exported to an Excel spreadsheet, where the final filtered object values are obtained for each image to use for cell loss graphs.

Due to there being 9 images per well for each treatment group replicate, the filtered object values obtained from CellProfiler for each region of the well must initially be added together to give a single value per well. Each replicate is then added together and averaged to derive one value per treatment group, for 2, 4 and 24 h. Each value per treatment group is then calculated as percentage of cell loss by treatment value/control value * 100%, keeping the control values at 100% (control value/control value * 100%). GraphPad Prism (version 10.1.2) was used to generate cell loss graphs as a percentage of cell loss for each of the three time points.

### 2.8. Statistical Analysis of Data

R (Version 4.4.2, R Foundation for Statistical Computing, Vienna, Austria), RStudio (version 1.1.414, RStudio, Inc., Boston, MA, USA) and the vascr package (Version 1.0.0, unpublished) were used to conduct a two-way analysis of variance followed by Tukey’s range test. All calculations were performed on unnormalized data, and p values were calculated relative to a media-only control at the same time point. Where not otherwise stated, significance was calculated at the final time point. Normality was confirmed using a visual inspection of the data and the Shapiro–Wilk normality test, whilst Levene’s test was used to verify homogeneity of variances. All graphs were generated using GraphPad Prism (version 7.03, La Jolla, CA, USA).

Statistically significant differences comparing experimental data and controls are indicated by asterisks. The *p*-value = 0.05 (*), 0.01 (**), 0.001 (***), 0.0001 (****). No asterisks = no statistical significance.

## 3. Results

### Killing of Glioblastoma Cultures with NK-92 Cells

Using NK-92 cells, we conducted the killing assays across multiple different patient-derived glioblastoma cultures that were originally developed by Auckland Cancer Society Research Centre from patient tumour resections. These cultures were NZB11, NZB12, NZB14 and NZB15 as detailed previously [26,27,28]. For these killing assays, we developed an imaging pipeline that was enhanced to discriminate adherent NK-92 cells (CD45 expression) from the glioblastoma cells (nuclei size and actin staining), in order to quantify the number of surviving cells.

**Figure 1:**
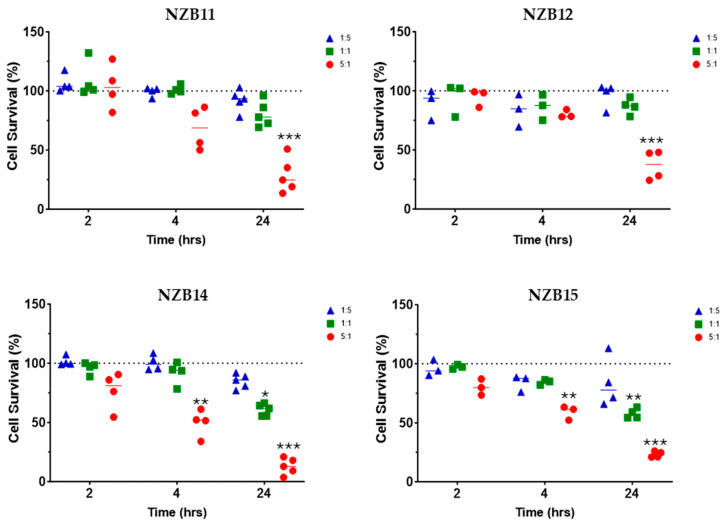
NK-92 mediated glioblastoma cell loss. Imaging-based quantification of glioblastoma cell loss following co-culture with NK-92 cells. NK-92 cells were added at ET ratios of 1:5, 1:1 and 5:1 as indicated in the colour coded key. Data show glioblastoma cell loss for four different patient-derived glioblastoma cultures (NZB11, NZB12, NZB14 and NZB15). Each independent experiment was conducted 3–5 times. Each dot on the graph represents the average cell loss calculated from 27 to 36 images per treatment in a single experiment (see Methods section). The 100% dotted line represents the control glioblastoma cell counts. Thus, 25% “surviving” cells means 75% of the glioblastoma cells have been lost. Statistical comparisons were conducted (see Methods) with the treatment group and control group. Significance is shown where the *p*-value = 0.05 (*), 0.01 (**) and 0.001 (***).

An advantage of using the NK-92 platform is that the NK-92 cells are reported to have lower inhibitory receptor expressions than their primary NK counterparts. We therefore used a range of ET ratios (1:5, 1:1 and 5:1) that was based on our earlier observations with primary blood NK cells (see Supplemental Figures S5 and S6). We predicted to see glioblastoma cell loss at the high 5:1 ratio, with the hope of seeing convincing glioblastoma cell loss at the lower ratios too. Figure 1 summarises some of these data, which show the collation of glioblastoma cell loss across a range of independent experiments for each patient-derived glioblastoma line. Each dot in the graph represents an independent experiment, from 3 to 5 independent experiments. As predicted, we saw consistent and impressive glioblastoma cell loss at the high ET ratio of 5:1, which was greatest 24 h after NK-92 addition. More impressively, we saw significant glioblastoma cell loss at the 1:1 ratio for NZB15 and NZB14 of around 30–45% across the independent experiments 24 h after addition of the NK-92 cells. There was also a trend of an effect at the lowest ratio of 1:5 for both these glioblastoma cultures, but this ratio was not significant statistically. Across these experiments, it was only at the 5:1 ratio where we lost most of the glioblastoma cells, which was evident at the 24 h time point. This also meant that even in the 5:1 ratio, which is a relatively high ratio, substantial numbers of glioblastoma cells were resistant to the NK-92 cells and survived. It is worth emphasising that as glioblastoma cells are lost, the ET ratio increases in favour of the NK-92 cells.

As detailed in the methods, the high-content image analysis pipeline was specifically developed to quantify the number of surviving glioblastoma cells post exposure to the NK-92 cells. It is important to highlight that the values represented in the graph for each dot are from 9 images per well and where each individual treatment would be carried out across 3–4 wells. Therefore, the mean percentage values are equated from at least 27–36 images per treatment for every independent experiment, thus providing higher confidence in the measured cell counts.

**Figure 2:**
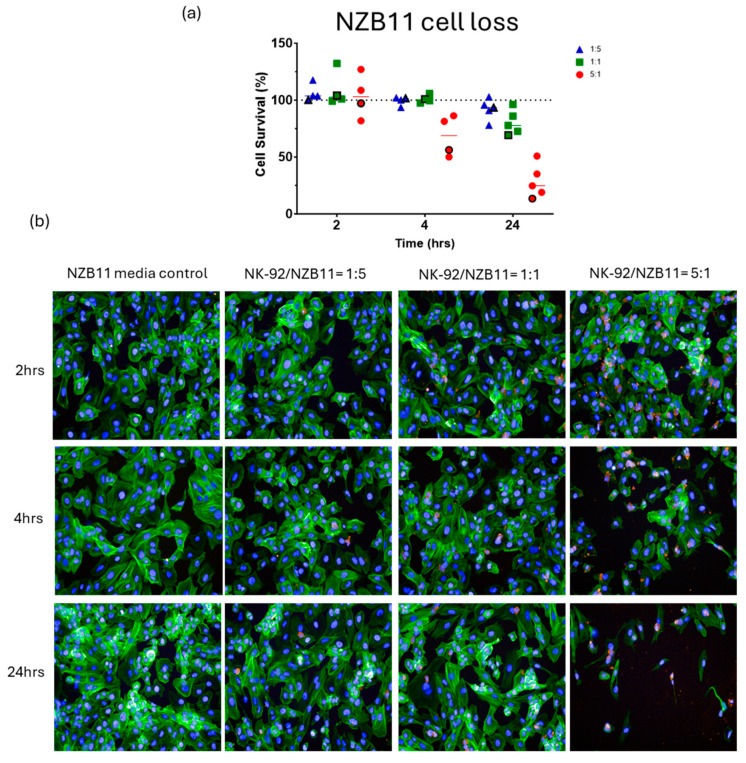
**NBZ11 glioblastoma cell loss.** Imaging-based quantification of glioblastoma cell loss following co-culture with NK-92 cells. NK-92 cells were added at ET ratios of 1:5, 1:1 and 5:1 as indicated in the colour coded key. The images are from a single representative experiment in the series, which is identified in the quantification graph. The symbol in (**a**) is highlighted with a black outline. (**b**) In these images the glioblastoma cells are green (actin), and the NK-92 cells are red. Nuclei are counterstained with Hoechst. Note the general lack of adherent NK-92 cells (red). Time represents the duration of co-culture for each respective E:T ratio.

**Figure 3:**
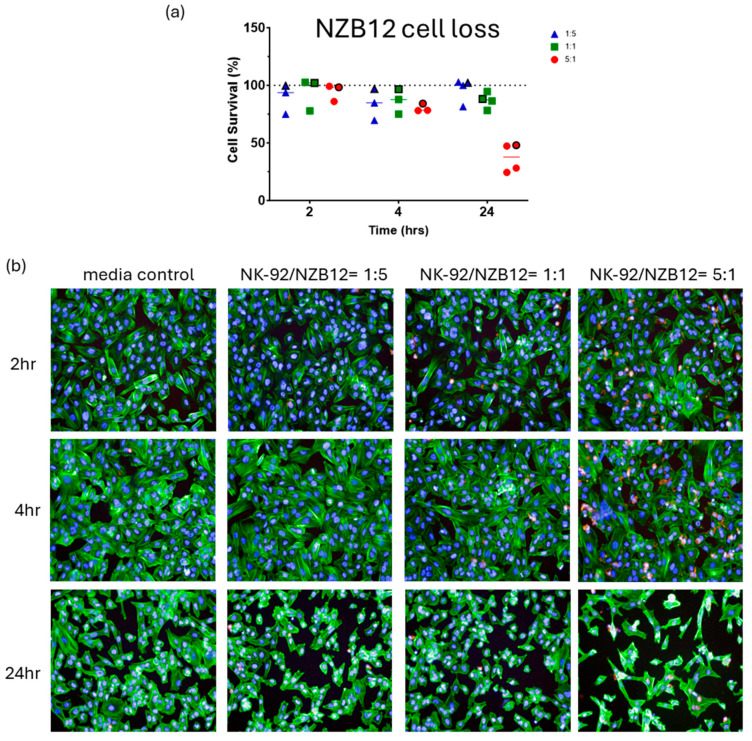
**NBZ12 glioblastoma cell loss.** Imaging-based quantification of glioblastoma cell loss following co-culture with NK-92 cells. NK-92 cells were added at ET ratios of 1:5, 1:1 and 5:1 as indicated in the colour coded key. The images are from a single representative experiment in the series, which is identified in the quantification graph, where the symbol in (**a**) is highlighted with a black outline. (**b**) In these images the glioblastoma cells are green (actin), and the NK-92 cells are red. Nuclei are counterstained with Hoechst. Time represents the duration of co-culture for each respective E:T ratio.

**Figure 4:**
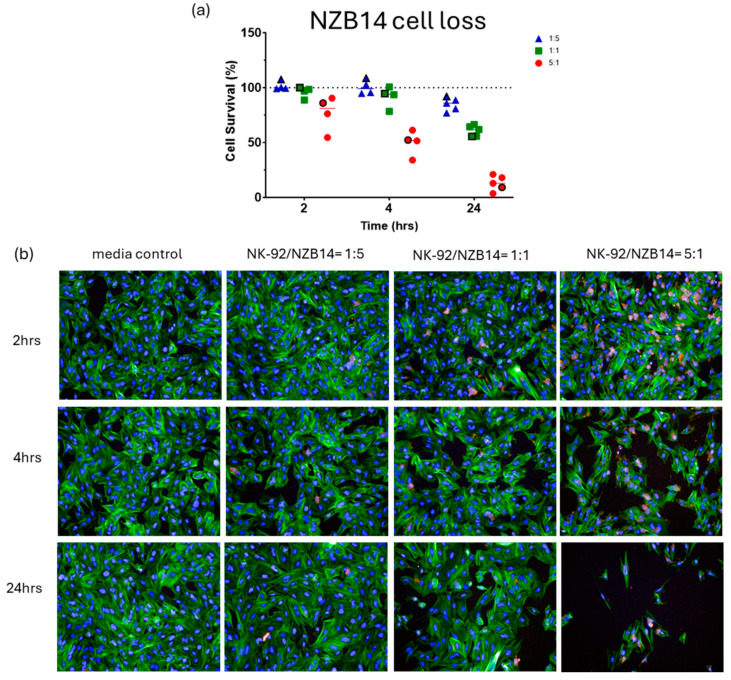
**NBZ14 glioblastoma cell loss.** Imaging-based quantification of glioblastoma cell loss following co-culture with NK-92 cells. NK-92 cells were added at ET ratios of 1:5, 1:1 and 5:1 as indicated in the colour coded key. The images are from a single representative experiment, which is identified in the quantification graph, where the symbol in (**a**) is highlighted with a black outline. (**b**) In these images the glioblastoma cells are green (actin). Note the highly variable levels of actin intensity across the NZB14 culture. The NK-92 cells are red. Note the greater adhesion of NK-92 to the NZB14 culture, especially at 2 h post addition. Nuclei are counterstained with Hoechst. Time represents the duration of co-culture for each respective E:T ratio.

**Figure 5:**
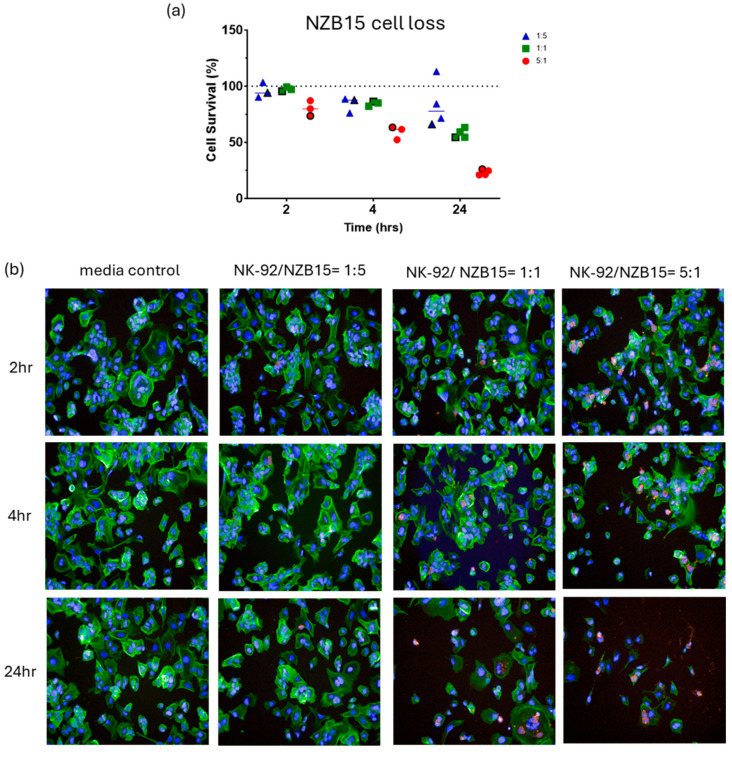
**NBZ15 glioblastoma cell loss.** Imaging-based quantification of glioblastoma cell loss following co-culture with NK-92 cells. NK-92 cells were added at ET ratios of 1:5, 1:1 and 5:1 as indicated in the colour coded key. The images are from a single representative experiment, which is identified in the quantification graph, where the symbol in (**a**) is highlighted with a black outline. (**b**) In these images the glioblastoma cells are green (actin). Note the highly variable NZB15 morphology and clustering. The NK-92 cells are red. Nuclei are counterstained with Hoechst (blue). Time represents the duration of co-culture for each respective E:T ratio.

Figure 2, Figure 3, Figure 4 and Figure 5 show representative images from these experiments. The NK-92 cells were identified with CD45 expression (red), and the glioblastoma cells are identified with phalloidin-green to stain their extensive cytoplasmic actin and Hoechst for their large nuclei. There is clear evidence of the loss of the glioblastoma cells at the 5:1 ratio and in the respective 1:1 ratio treatment. Also evident is the apparent lack of NK cell adhesion in most of these images especially for the 1:1 and 5:1 ratios, where we would expect to see lots of adherent NK-92 cells. This perhaps suggests that adhesion is weak and does not survive the fixation or staining protocols, or that the NK-92 adhesion is transient.

As these data strongly suggest glioblastoma cell loss by the NK-92 cells, we next employed ECIS technology to investigate the temporal kinetics of the global impact of the NK-92 cells on the glioblastoma cultures. ECIS technology is a real-time biosensor platform that measures the relative adhesion of cells [27,29,30,31,32,33,34,35,36,37]. In this scenario, it is the adhesion of the glioblastoma cells [27] that is measured directly (target cells) and thus any changes to the glioblastoma cell number or strength of adhesion will alter the total adhesion measured. In this context, a loss of glioblastoma cells will result in a reduction in overall adhesion strength (measured by overall resistance (R)). In addition, cellular shrinkage and cell compromise will also affect total adhesion and will also reduce total adhesion strength.

Supplemental Figure S7 shows the baseline adherent properties of each of the glioblastoma cultures over the initial 96 h of culture. The relative adhesion strength of each culture is indicated by the resistance (ohms; Ω) value, where the larger the resistance value the greater the overall strength of adhesion of the cells. Generally, the adherent properties of the NZB11 cultures and the NZB14 cultures are stronger than the NZB12 or NZB15 when seeded at identical starting numbers (10,000 cells per well), whereas the resistance values shown in Supplemental Figure S7 for the NZB19 and NZB13 lines were from 20,000 cells per well and were not high enough to proceed further.

**Figure 6:**
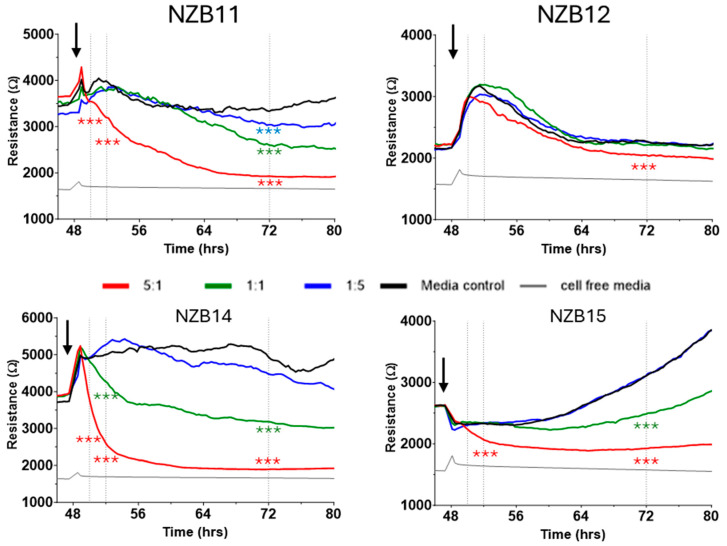
**Real-time biosensor-based assessment of NK-92 effects on the glioblastoma cultures.** ECIS biosensor technology measures the relative adhesion strength of the glioblastoma culture and indicates when the adherent behaviour of the glioblastoma cells changes. The resistive value is a measure of the glioblastoma adhesion across the ECIS arrays, where stronger net adhesion results in greater resistance. The grey line is the resistance in the absence of cells referred to as the cell-free resistance (around 1600–1700 ohms). NK-92 cells were added to glioblastoma cultures at ~48 h post seeding into ECIS 1E+ plates, as indicated by the black arrows. The greater the reduction in resistance following the addition of the NK-92, the more cellular adhesion has been lost. The temporal nature of ECIS reveals whether the loss in adhesion is sustained or transient, where sustained loss is consistent with cell compromise and death. These data are from independent experiments, representative of at least 3–4 independent experiments. Statistical comparison was conducted at the time points indicated by the vertical dotted lines representing 2 h, 4 h and 24 h post addition of the NK-92 cells. Statistical significance is shown where the *p*-value = 0.001 (***).

Figure 6 shows representative ECIS graphs for each glioblastoma culture. These graphs show responses that are representative of at least four independent ECIS experiments. As expected, there is a pronounced reduction in adhesion (resistance) for NZB11, NZB14 and NZB15 following the addition of the NK-92 cells at an ET ratio of 5:1. This loss in resistance indicates an overall loss of adhesion due to glioblastoma compromise and death, consistent with the imaging conducted previously. The greatest effect is observed with the NZB14 cultures, where the loss in adhesion was immediate and sustained (at 5:1 ratio). NZB14 also showed the greatest effect at the lower ET ratio of 1:1. Interestingly, the NZB11 and NZB15 cultures showed a measurable loss in adhesion for the 1:1 ratio, however that effect was delayed and was observable some 8–16 h after the NK-92 addition. Interestingly, ECIS was unable to confidently reveal much of a difference across the different ET ratios for the NZB12 cultures.

One of the most important aspects of ECIS is the real-time or temporal nature of the measurements. The gross reduction in the glioblastoma cell adhesion (loss of ECIS resistance) could be due to cell death, cell shrinkage or a reduction in basolateral focal adhesion of cells. In our experience, sustained loss in adhesion is more consistent with cellular compromise and cell death [27,30,34]. Therefore, next we looked at the images of the early time points for evidence of glioblastoma cell compromise and death.

**Figure 7:**
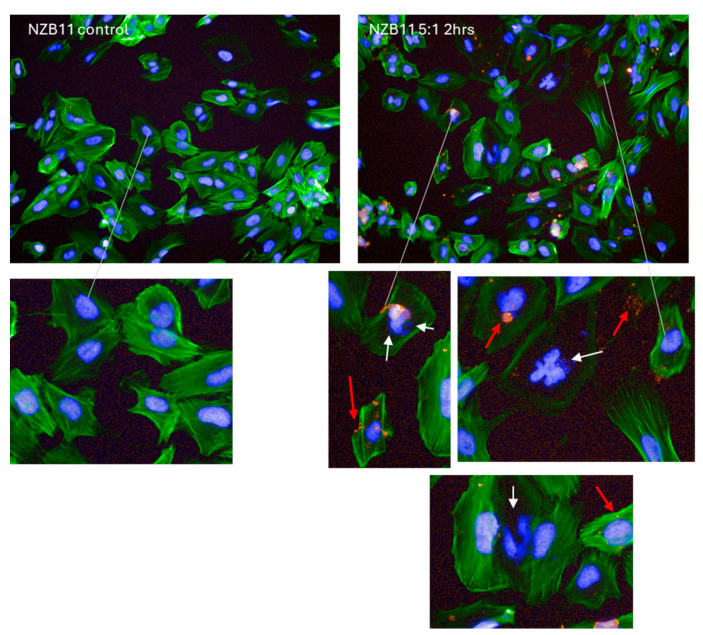
**Evidence of NZB11 glioblastoma cell death.** Images from NZB11 cultures two hours after NK-92 cell addition. Control glioblastoma cells are shown in the left panels where glioblastoma cells are green with Hoechst-stained nuclei; note the uniform nuclei and intact actin structures. In the right-side panels, the white arrows point to abnormal NZB11 nuclei, and the red arrows point to CD45-positive puncta and debris from the NK-92 cells. Karyorrhectic nuclei are evident, indicative of early signs of glioblastoma cell death. These images were acquired 2 h after NK-92 addition.

**Figure 8:**
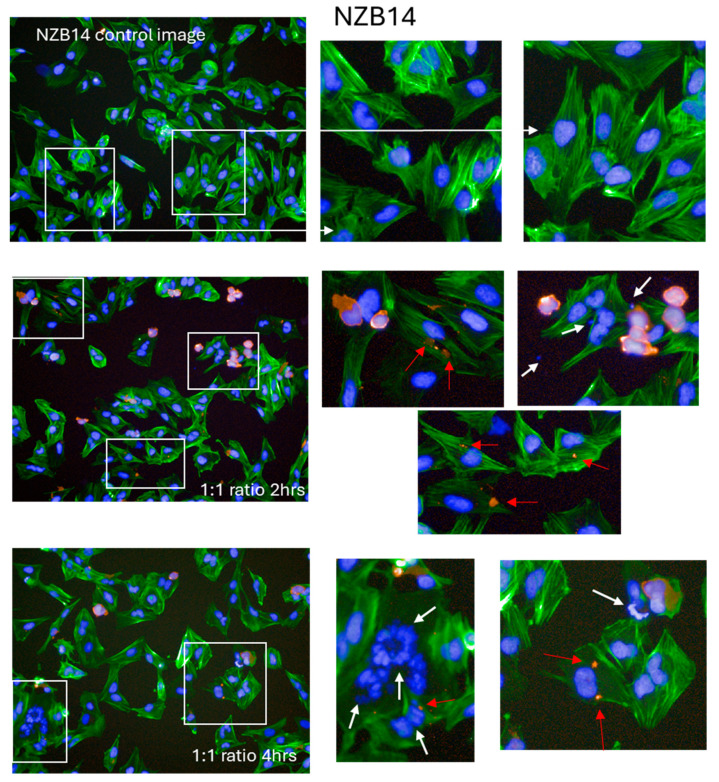
**Early evidence of NZB14 glioblastoma cell compromise.** Images from NZB14 cultures 2–4 h after NK-92 addition. Control glioblastoma cells are shown in the top panels where glioblastoma cells are green with Hoechst-stained nuclei. Note the uniform nuclei and intact actin structures. The lower panels show NK-92 images from 1:1 ratio at 2 h and 4 h, respectively. CD45 staining (red) indicates the NK-92 cells. The white arrows point to abnormal NZB14 nuclei, and the red arrows point to CD45 positive puncta and debris from the NK-92 cells. After 2–4 h, many of the glioblastoma cells have abnormally shaped nuclei that appear karyorrhectic and in small puncta. This is indicative of early signs of glioblastoma cell death. These images were acquired 2–4 h after NK-92 addition.

**Figure 9:**
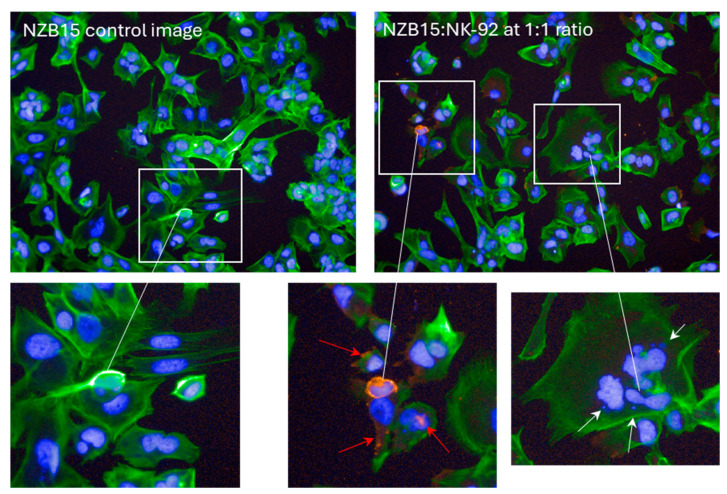
**Early evidence of NZB15 glioblastoma cell compromise.** Images from NZB15 cultures 2 h after NK-92 addition (1:1 ratio). Control glioblastoma cells are shown in the top left panel where glioblastoma cells are green with Hoechst-stained nuclei. CD45 staining (red) indicates the NK-92 cells. The white arrows point to abnormal NZB15 nuclei, and the red arrows point to CD45 positive puncta and debris from the NK-92 cells. Numerous glioblastoma cells have abnormally shaped nuclei that appear karyorrhectic and adjacent small DNA puncta. This is indicative of early signs of glioblastoma cell death. These images were acquired 2 h after NK-92 addition.

**Figure 10:**
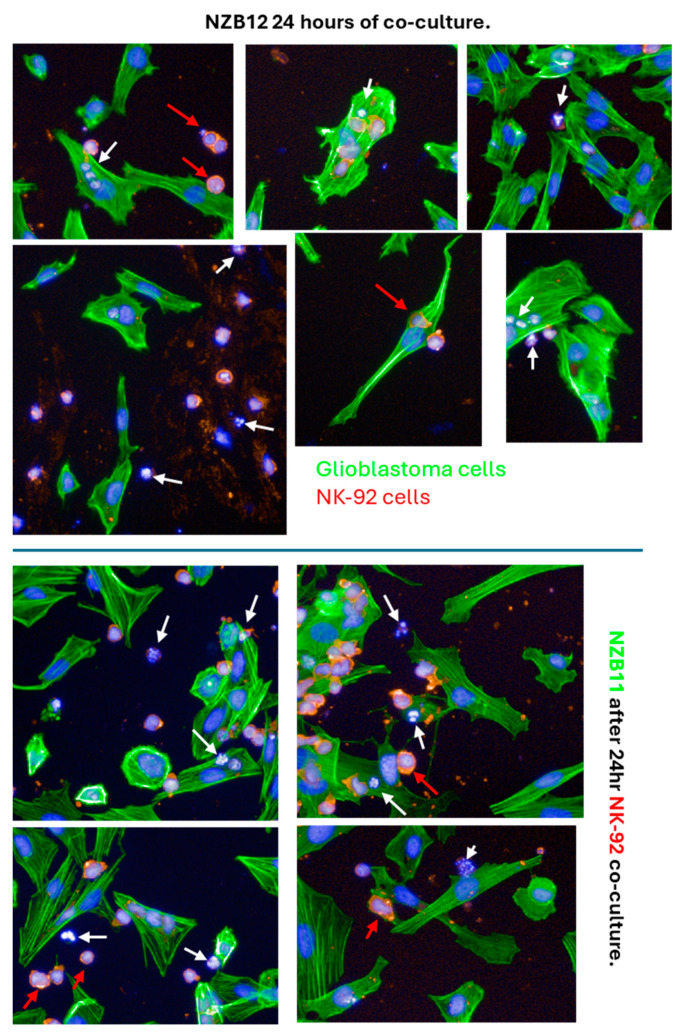
**Evidence of NK-92 cell-death 24 h after their addition to the glioblastoma cultures.** The NK-92 cells are counterstained with CD45 (red). Some healthy/intact NK-92 cells are evident and highlighted with the red arrows. However, there are numerous examples of NK-92 cells with abnormal disintegrated nuclei, highlighted by the white arrows. These nuclei are indicative of NK-92 cells that are dying. There is also a considerable amount of CD45 stained puncta (red), which may be from dead NK-92 cells. The glioblastoma cells are counterstained green with Actin Ready probes. These images were acquired 24 h after NK-92 addition.

There was considerable evidence of glioblastoma cell compromise at these early time points (2–4 h) and 24 h after NK-92 addition. These are shown in Figure 7, Figure 8, Figure 9 and Figure 10. Figure 7 shows examples of early compromise to the NZB11 glioblastoma cells mediated by NK-92 cells within 2 h. The righthand panels reveal abnormal nuclei in some glioblastoma cells indicated by the white arrows. These are consistent with the morphology of multi-lobular apoptotic or karyorrhexic nuclei, indicating compromised cells. Some of these glioblastoma cells have abnormal or no actin cytoskeleton, which is also an early sign of loss of integrity.

In Figure 8 and Figure 9, we see examples of intact adherent NK-92 cells attached to the glioblastoma cells (NZB14 cells). We also highlight that at 2–4 h after the NK-92 addition, the presence of CD45 positive puncta attached to or inside the glioblastoma cells (red arrows). These are smaller than an intact nucleated NK-92 cell, and in some glioblastoma cells we observed numerous red puncta. These are potentially left behind by previously attached NK-92 cells or may represent material injected by the NK-92 cells into the glioblastoma cells. In Figure 9, which features the NZB14 glioblastoma cells, examples of nuclei that appear to be disintegrated and abnormal (white arrows) are evidence of the glioblastoma cells dying. Figure 10 also shows evidence of abnormal glioblastoma nuclei (NZB15; white arrows), again with abnormal actin cytoskeletal structures, following 2 h treatment with the NK-92 cells. This is strong evidence of early compromise and the loss of integrity of the glioblastoma cells within 2–4 h of NK-92 attack. NZB15 glioblastoma cells typically grow in clumps and often on top of each other, and this makes the high-content quantification more challenging but would also likely affect the accuracy of the ECIS measurements in terms of measuring cell loss. This is an important consideration as cultures that prefer to grow in 3D clumps will potentially provide less coverage of the ECIS electrode arrays, highlighting the importance of multiple measurements of cell loss and compromise, as we have conducted here.

In the high-content imaging experiment and ECIS experiments, we were never able to kill 100% of the glioblastoma culture. We were at best able to achieve 80–90% glioblastoma cell loss in our most successful experiments. Although this is very promising, realistically this was only at the high ET ratio of 5:1. This also means that at 1:1 ratio, even at 24 h of killing, it was only a minority of the glioblastoma cells that the NK-92 line was able to kill as most glioblastoma cells survived. This inevitably means that these cultures have subsets of glioblastoma cells that are particularly resistant or adaptive to the NK-92 cells. Also, as we kill and lose glioblastoma cells, the resultant ET ratio increases in favour of the NK-92 cells. However, even with this advantage some glioblastoma cells are capable of evading NK-92 killing.

**Figure 11:**
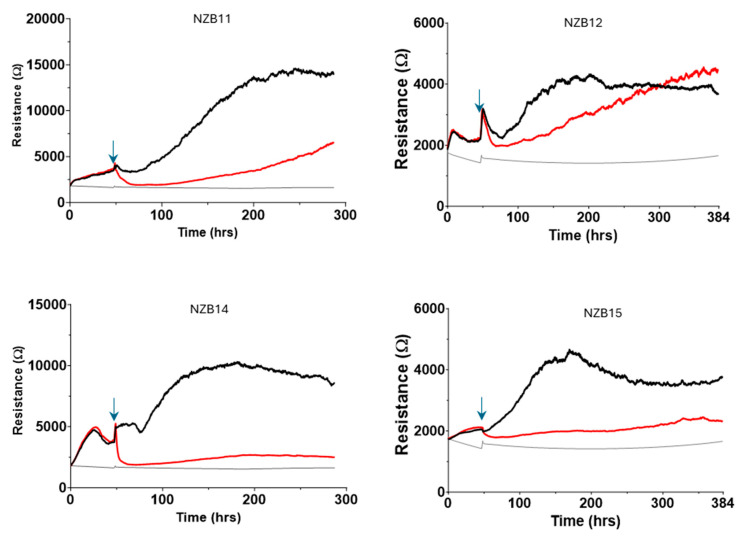
**Long-term ECIS experiments demonstrate regrowth of the glioblastoma cultures.** ECIS biosensor experiments were conducted over 300–400 h to see whether the surviving glioblastoma cells were capable of regrowth. Data show the regrowth of the glioblastoma cells post addition of NK-92 cells at the 5:1 ratio (red curve), which resulted in the initial loss of most of the glioblastoma cells. Some regrowth was observed for all cultures. The time of NK-92 addition is indicated by the blue arrow. The black curve is the control media treated glioblastoma cells. These longer-term experiments are representative of 3 independent repeats.

We therefore set up long-term ECIS experiments to look across a 300–500 h time frame to see what happens to the glioblastoma cells that survive the initial NK-92 killing phase. Very interestingly, we see a slow progressive regain in ECIS resistance for each of the cultures (Figure 11). This indicates an increase in net adhesion, which we hypothesised was due to increased glioblastoma cell numbers, thus increased total adhesion. Regrowth for the NZB11 and NZB12 cultures was typically faster and more complete than either NZB14 or NZB15 based on the ECIS curves.

**Figure 12:**
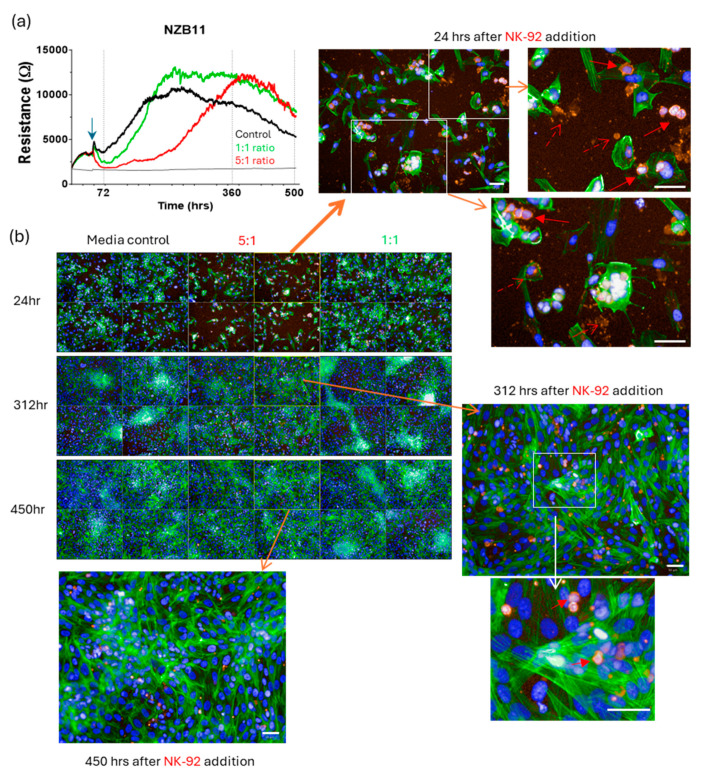
**Assessment of long-term regrowth from surviving glioblastoma cells following NK-92 mediated killing.** On the ECIS graphs (**a**) the time scale is from time 0 and NK-92 cells were added 48 h into the culture. (**b**) Cultures were assessed at designated time points to visualise the glioblastoma cell regrowth. This was 24 h, 312 h and ~450 h after NK-92 addition. Glioblastoma cells were stained with actin-green and nuclei with Hoechst. NK-92 cells are red (CD45 positive). Following the initial glioblastoma loss, substantial regrowth is evident. In the zoom images, the solid red arrows indicate intact NK-92 cells, whereas the dashed red arrows indicate NK-92 debris. White scale bars represent 50 µm.

**Figure 13:**
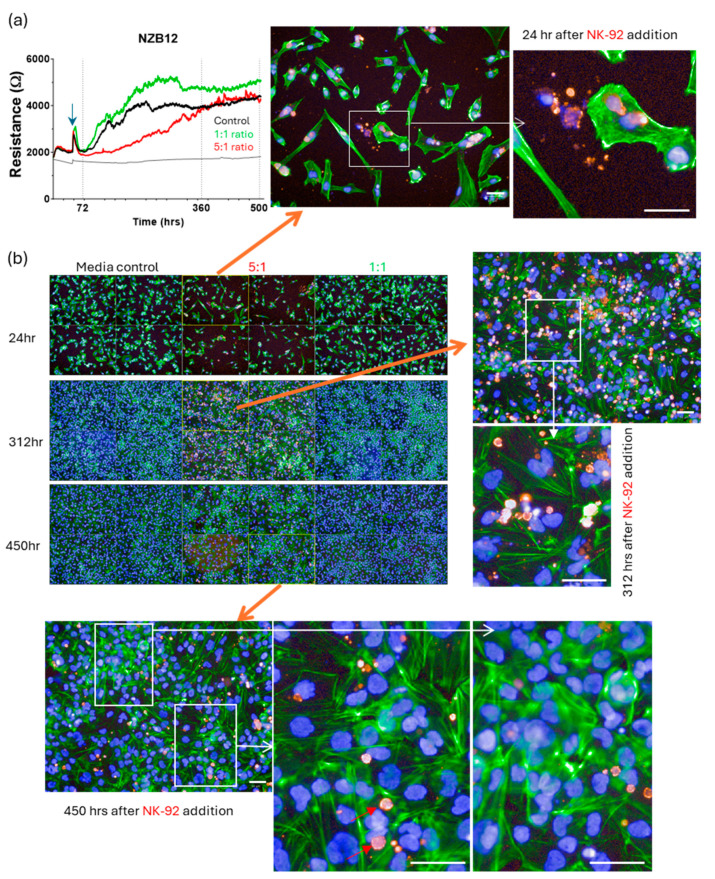
**Regrowth of NZB12 from surviving glioblastoma cells following NK-92 mediated killing.** (**a**) The ECIS graphs indicate NZB12 regrowth within ~300 post NK-92 addition. (**b**) Cultures were assessed at 24 h, 312 h and ~450 h after NK-92 addition. Glioblastoma cells were stained with actin-green and nuclei with Hoechst. NK-92 cells are red (CD45 positive). Note the abundance of the CD45 positive NK-92 puncta still present in the 312 h images. Many are not intact viable NK-92 cells and are gone by 450 h. Imaging panels support this observation for the 5:1 and 1:1 ratios. Following the initial glioblastoma loss, substantial regrowth is evident. White scale bars represent 50 µm.

**Figure 14:**
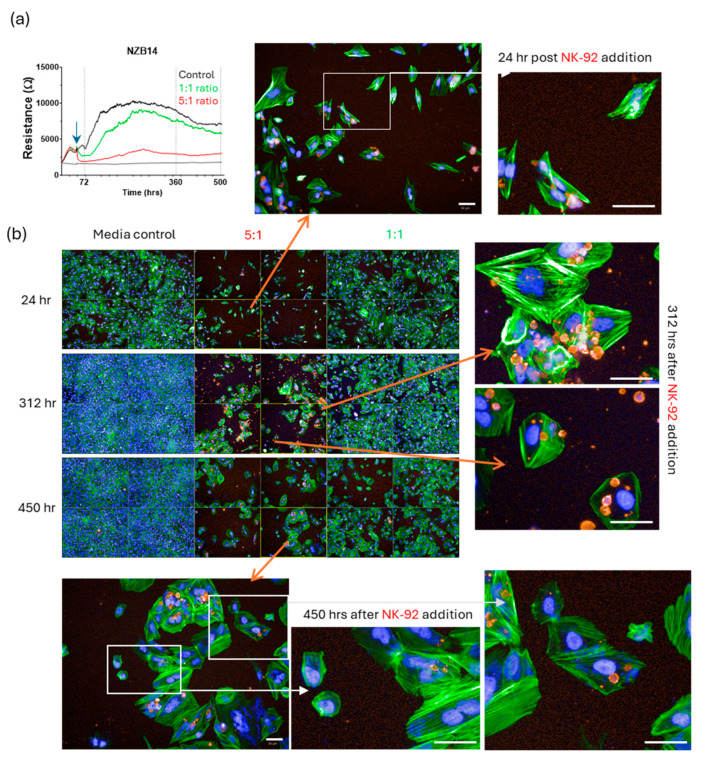
**Partial regrowth of NZB14 from surviving glioblastoma cells following NK-92 mediated killing.** (**a**) The ECIS curves indicate modest NBZ14 regrowth and the imaging panels support the ECIS data. (**b**) Cultures were assessed at 24 h, 312 h and ~450 h after NK-92 addition. Glioblastoma cells were stained with actin-green and nuclei with Hoechst. NK-92 cells are red (CD45 positive). Note the abundance of the CD45 positive NK-92 puncta present in the 312 h images, which are mostly gone by 450 h. White scale bars represent 50 µm.

**Figure 15:**
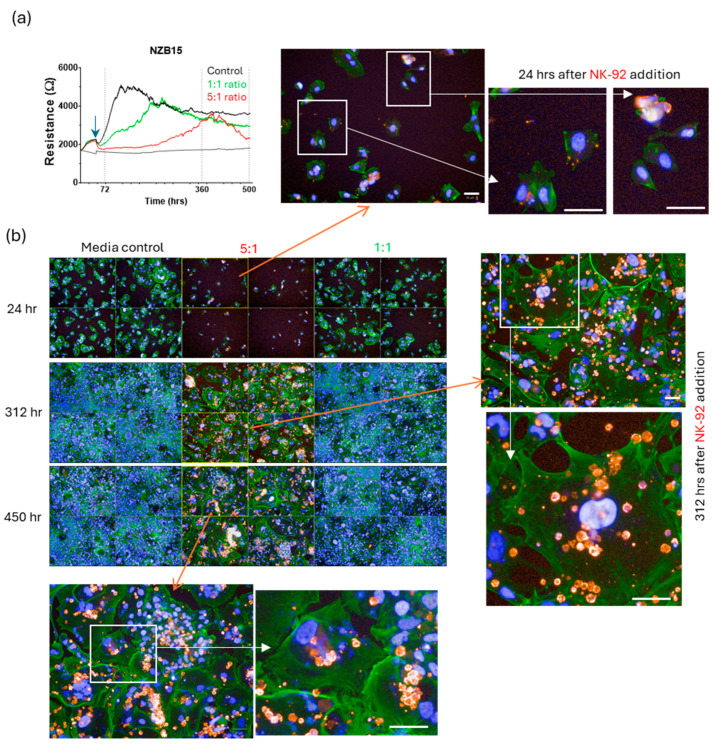
**Partial regrowth of NZB15 from surviving glioblastoma cells following NK-92 mediated killing.** (**a**) The ECIS curves indicate slow NBZ15 regrowth following 5:1 NK-92 killing. (**b**) Cultures were assessed at 24 h, 312 h and ~450 h after NK-92 addition. Glioblastoma cells were stained with actin-green and nuclei with Hoechst. NK-92 cells are red (CD45 positive). The imaging panels support the ECIS data. Note the very large NZB15 glioblastoma cells present in the zoom panels. Note the abundance of the CD45 positive NK-92 puncta present in the 312 h images, which are still present at 450 h. White scale bars represent 50 µm.

Imaging from these longer-term cultures (Figure 12, Figure 13, Figure 14 and Figure 15) very clearly reveals regrowth of the glioblastoma cells from the time of maximal observed glioblastoma cell loss (~24 h post addition of NK-92 cells). We have focussed on the regrowth of the 5:1 killing experiments, as the 1:1 cultures were comparable to the controls at the >300 h time points. However, the images for the 5:1 co-culture reveal evidence of different unexpected events occurring in these cultures in addition to the regrowth. Consistent with the ECIS data, considerable regrowth was observed for NZB11 and NZB12, whereas regrowth for NZB14 and NZB15 was partial (Figure 12, Figure 13, Figure 14 and Figure 15). Very interesting, some intact NK-92 cells are evident in the 24 h images; however, most of the CD45 staining pertains to debris of compromised-looking NK-92 cells. This implies that the NK-92 cells quickly become exhausted or suppressed and lose killing potential. It was also surprising to observe so much NK-92 debris 300–450 h after their killing in some cultures (NZB15 and NZB12). In contrast, the late time point (450 h) revealed very few intact NK-92 cells or related debris in the NZB11 or NZB14 cultures by comparison.

Importantly, these data demonstrate that even with small numbers of surviving glioblastoma cells, there were sufficient surviving glioblastoma cells with proliferative capacity to successfully reseed the culture, even in the cultures that were most sensitive to the NK-92 killing. This is consistent with what would occur post-surgery in most patients where the resistant glioblastoma clones regrow, reseeding the tumour resulting in tumour relapse.

## 4. Discussion

Outcomes for glioblastoma patients are abysmal where the median overall survival has hardly increased in the last 20 years. This is evidenced by the fact that if untreated, survival is only 3–4 months and the best possible clinical care (Stupp’s protocol; [3]) may only provide an extra 6–12 months for most patients.

In this research, we asked whether we could kill glioblastoma cells using Natural Killer cells. Initially, we used NK cells from the blood of healthy donors; however, this was incredibly problematic, and we saw highly variable degrees of sensitivity to the donor NK cells (see Supplemental Figures S5 and S6). A major issue was the yield of primary NK cells, which limited the range of experiments we could conduct. Additionally, our institutional human ethics only allowed for blood collections from de-identified healthy persons. We were therefore unable to go back to the same donor to obtain more blood to conduct repeat experiments. These are known issues for those working with less abundant primary leukocyte populations and restricted ethics. We therefore obtained the NK-92 cell line, which is an IL-2 dependent NK line derived from a patient with aggressive NK-cell lymphoma by Hans Klingemann [17,18,24] in 1992. Since then, this NK line has shown impressive killing of a range of tumour cell types [19,20,23] and is now entering phase I and phase II clinical trials for various haematological cancers [24,25,38]. The enhanced killing phenotype of the NK-92 line is partially explained by the fact the cells are activated with IL-2 and they lack or have lower expression of various NK inhibitory receptors, thus reducing the ability of the target tumour cells to suppress them [19,23,39].

In our first series of NK-92 killing assays, we developed a high-throughput and high-content image analysis pipeline to enable accurate quantification of glioblastoma cell loss following exposure to the NK-92 cells. The robustness of this assay is a function of it using at least 3–4 replicate wells per treatment and then acquiring at least nine images from within the same well using an automated high-content fluorescent imaging system. This means that for every individual treatment we quantify glioblastoma cell numbers for 27–36 images, and then each independent experiment is repeated at least three times for consistency. Thus, the data shown in Figure 1 are from over 5000 images that have been acquired and quantified in a non-biased manner. This assay counts the number of glioblastoma cells that survive and thus indicates the number of glioblastoma cells that have been lost. Where the NK-92 cells have no effect and show no cytotoxicity, there is no loss of glioblastoma cells. However, where there is loss of the glioblastoma cells there are several pragmatic possibilities that could explain this loss, the most obvious being NK-92 mediated killing. The second is where the NK-92 cells provoke the glioblastoma cells to detach from the substrate and are lost and assumed dead. It is possible to conclude that these data show that most of the glioblastoma cells survive the NK-92 killing period for at least 24 h at ET ratios of 1:1 and 1:5, and it is only at the higher 5:1 ratio where we see the majority of the glioblastoma cells have sensitivity to the NK-92 exposure.

To better understand the dynamics of glioblastoma cell compromise and sensitivity to the NK-92 we conducted the killing assays and monitored the health of the glioblastoma cells using ECIS technology. This was conducted in conjunction with imaging to assess changes in glioblastoma cell morphology and identify evidence of the glioblastoma cell dying. As detailed extensively in [27,40,41,42], ECIS is a real-time biosensor technology that can monitor changes in cell health and viability [43,44] based on the adherent nature of the cells. Glioblastoma cells are reasonably adherent, and this assay shows good application [27] and detection of the loss of adhesion temporally for several of the glioblastoma lines. The most powerful observation from this technology is the temporal nature showing how fast the glioblastoma cells respond to the NK-92 cells and start to lose adhesion. This is especially obvious for the 5:1 ET ratio. What is most important here is that this loss is immediate and permanent. If the glioblastoma cells simply detached and were still alive, we would expect re-attachment within several hours, depicted as transient loss in resistance rather than sustained loss. As this does not happen, the major conclusion from these ECIS observations is that the glioblastoma cells are compromised, and death is occurring. Like the prior imaging ECIS, this also reveals that the loss of adhesion is not maximal and the red curves (5:1 ratio) never reach the cell-free point, which reflects zero cell adhesion. This means there are always surviving glioblastoma cells, precisely as we see in the imaging.

Further, we show morphological compromise in the target glioblastoma cells early in the killing assays consistent with the loss of cells and loss of adhesion. The imaging reveals numerous glioblastoma cells with aberrant nuclei that are disintegrating, blebbing, condensed and some also have micro-nuclear blobs, each of which are consistent with apoptotic morphologies and karyorrhectic nuclei [45,46,47,48]. These glioblastoma cells are destined to die. These were readily observed at 2–4 h in all cultures exposed to NK-92 cells at the 1:1 and 5:1 ratios. Notably, many of these compromised glioblastoma cells also have aberrant actin structures, cytoskeletal shrinkage or very little cytoplasmic actin that was visible. Collectively, these are all consistent with cell compromise where these glioblastoma cells are in early stages of death.

Curiously we also observed an interesting stain pattern from the NK-92 cells. Specifically, this relates to CD45-positive puncta from the NK-92 cells, which we speculate is potentially from material injected into the glioblastoma cells by the NK-92 cells or represents cell membranes left behind after the NK-92 cell has detached. The glioblastoma cells do not express CD45 and so this staining can only be derived from the NK-92 cells. As the main mechanism of killing is via NK-92 membrane attachment and injection of lytic enzymes, this staining pattern may reveal remnants of where the NK-92 cells were in the context of attempting to kill the glioblastoma cells. This observation is intriguing and requires further investigation in future studies.

Our most critical observation was that although we could certainly kill some glioblastoma cells and perhaps the majority at the 5:1 ratio, we were never capable of killing 100% of the culture. This means that there were always clones of glioblastoma cells with greater resistance to the NK-92 cells that were able to evade killing even when the NK-92 ratio increases. This observation was eloquently highlighted in the long-term ECIS experiments conducted over >300–500 h, where we see slow regrowth of the glioblastoma cultures over the 3–4-week period. This shows that the small number of surviving glioblastoma cells are capable of reseeding the culture and we have previously shown using serial dilution assays that we can grow glioblastoma tumour spheres from single diluted cells which also required 3–4 weeks to grow [26,28]. One of the reasons for the very poor outcomes and the recurrent nature of glioblastoma post-surgery is the fact that the tumour initiating cells exist in the surrounding surgical margins. Cells that survive in the patient cause the resultant tumour regrowth. This means that an effective therapy that provides hope for patients must kill all of the glioblastoma tumour cells as anything less than 100% will not be effective. The goal now is to identify the molecular targets expressed by the resistant glioblastoma cells that convey resistance to the NK-92 cells and identify mechanisms to increase NK-92 cell potency to enhance killing in ways to reduce the ET ratios and achieve 100% targeted glioblastoma cell death.

## 5. Future Directions

We are currently conducting an extensive molecular characterisation of the ligands and molecules used by the glioblastoma cells to suppress NK-92 mediated attack and thus suppress NK-92 killing activity. This is the essential next step in order to identify targets to enhance and drive NK-92 killing to achieve 100% maximal killing. Anything less than 100% glioblastoma cell death will not be good enough and only once that can be achieved will there be any chance for patients to see a treatment that offers hope for a cure.

## Figures and Tables

**Table 1:** Immunocytochemistry antibodies and stains used in the imaging pipeline.

Staining Target	Staining Dye/Antibody	CAT#	Dilution
NK-92	Mouse anti-CD45 antibody (Primary Antibody)	ab30470-100ug	1:400
	Anti mouse Alexa Fluor™ 594 (Secondary antibody)	A11005	1:400
Nuclei	Hoechst 33342 (nuclei/DNA)	H3570	1:10,000
Glioblastoma cells	ActinGreen™ 488 ReadyProbes™ (AlexaFluor™ 488 phalloidin)	R37110	1 drop per mL of buffer

Table details staining details for the immunocytochemistry pipeline used to detect the NK-92 cells and glioblastoma cells.

## Data Availability

Access to original data can requested by contacting the corresponding author Associate Professor E Scott Graham at s.graham@auckland.ac.nz.

## Supplementary Materials

The following are available online at https://www.mdpi.com/article/10.3390/cells14010053/s1. Figure S1: Images of NK-92 suspension culture at various stages of growth imaged under phase microscope after 96 h of culture. Figure S2: Schematic of primary NK cell isolation and usage. Figure S3: Gating strategy of NK cells for flow cytometry analysis. Figure S4: Primary NK cell purity analysis by flow cytometry. Figure S5: NK cell killing of NZB11 glioblastoma cells. Figure S6: Primary NK cell killing of NZB19 glioblastoma cells with different NK donors. Figure S7: ECIS cell adhesion curves for each glioblastoma culture.
