# Supplementary material for: Trying to Kill a Killer; Impressive Killing of Patient Derived Glioblastoma Cultures Using NK-92 Natural Killer Cells Reveals Both Sensitive and Highly Resistant Glioblastoma Cells
Source: Cells. 2025 Jan 5;14(1):53. doi: 10.3390/cells14010053 (PMC11720543; doi:10.3390/cells14010053)
Supplement: Supplementary file 1 [file cells-14-00053-s001.zip › cells-3364126 SI-main.pdf]

Supplemental methods section

Collection of healthy donor blood and preparation of primary NK cells.

Blood was collected from healthy donors under human ethics approval number 014035 (University of Auckland Human Participants ethics committee). All participants provided written consent and 30-50ml of blood was collected by a qualified phlebotomist from donors aged 20-40 and were used in a de-identified manner.

Whole blood was processed immediately for isolation of peripheral blood mononuclear cells (PBMC). The blood was separated using Leucosep tubes (cat# 227290, Greiner Bio-One) containing 15mL of Lymphoprep (cat# 1114545, Alere Technologies) below the porous barrier. The blood was centrifuged at 800x g for 15 minutes at room temperature, with gentle acceleration and breaking to ensure maintenance of the buffy coat. Once centrifuged, the buffy coat containing the PBMC was gently removed and washed several times in RPMI to remove any Lymphoprep. The final cell pellet was reconstituted in isolation buffer (Ca^{2+} and Mg^{2+} free PBS supplemented with 0.1% BSA and 2mM EDTA). At this juncture, a proportion of the PBMC were used for assessment of leukocyte yield, with the remainder used for NK isolation.

NK isolation was conducted using the Dynabeads™ Untouched™ Human NK Cell Kit (cat# 11349D, ThermoFisher). NK isolation was conducted as per the manufacturer's protocol where typically a starting yield of 40 million PBMC would produce 1-3 million NK cells. This protocol is shown in supplemental figure 2.

Glioblastoma killing assays using primary blood-derived NK cells.

The killing assay consisted of co-culturing IL-2 activated primary NK cells (effector cells, "E") and glioblastoma cells (target cells, "T") seeded in 96 well plates. To ensure the glioblastoma cells were firmly adhered, they were cultured for 3 days prior to addition of the primary NK cells. Primary human NK cells require activation for suitable killing of tumour cells and so this was achieved using IL-2 (5ng/mL) for 3 days. This was routinely checked using flow cytometry to confirm the yield of NK cells and good viability based on 7AAD exclusion (supplementary figure 3). The FSC and SSC plots also confirm activation by IL-2 indicated by the increase in NK cell size and increased expression of CD56 and CD16 by the primary NK cells (supplementary figure 4). Prior to addition to the glioblastoma cultures, the NK cells were spun down at 160 g for 10 minutes, and the supernatant was discarded. The NK cells were resuspended in fresh RPMI, counted and then prepared at the appropriate ET ratio for addition to the glioblastoma cells. Three E:T ratios were used for most killing assays: 5:1, 1:1, and 1:5. The same volume of RPMI was added to each well, including control wells. After the addition of NK cells, the cells were placed in the incubator for 4 or 24 hours to allow killing to occur. After 24 hours the surviving glioblastoma cells were fixed after the NK suspension media was removed and discarded. The cells were fixed using 4% PFA for 10 minutes followed by a series of washes using PBS containing 0.1% triton X100 (PBST) to permeabilise the cells for immunocytochemical staining.

Immunocytochemical stain of primary NK cells and glioblastoma cells

Post-fixation and permeabilization as detailed above, glioblastoma cells were counterstained with ActinGreen™ 488 ReadyProbes™ (Thermo Fisher, CAT# R37110) and all nuclei were counterstained with Hoechst 33342 (Invitrogen CAT# H3570) at a dilution of 1:10,000 in PBS for 20 mins at room temperature. Cells were washed with PBST once and stored in PBS at $4^{o}C$ light protected until imaging was conducted. Images were acquired with an EVOS FL auto fluorescent microscope using the FITC cube and DAPI cubes to acquire 10 non-overlapping regions of interests around the middle of each well. These images were then imported into CellProfiler to quantify the number of glioblastoma cells. It was possible to discriminate the primary NK cells from the glioblastoma cells on the basis of their small nuclei (3-5μm), lack of cytoplasmic actin staining and

intensity of Hoechst uptake by the primary NK cells. Primary NK cells have very small intensely stained nuclei, whereas glioblastoma nuclei are typically 15-20 μm in diameter. Fine tuning was conducted to ensure CellProfiler counts matched manual counts using randomly chosen images. Data were then represented as a percentage change to the media treated glioblastoma counts (no primary NK cells) which represented 100%.

Supplemental Results

Killing glioblastoma cells with allogeneic NK cells from healthy donors?

The data shown in supplementary figure 5 and 6, shows an example of extent of glioblastoma cell loss with primary NK cells from healthy blood donors. Glioblastoma cells were seeded at 2000 to 5000 cells per well where the IL-2 activated primary NK cells were used at ET ratios of 1:5, 1:1 and 5:1. The data shown in supplementary figure 5 highlights the killing of NZB11 glioblastoma cells using NK cells from 3 different donors. Glioblastoma cell loss was observed with primary NK cells from various donors, but consistently only at an ET ratio of 5:1, which immunologically is a high ET ratio. It is also worth noting that the extent of glioblastoma cell loss was usually less than 50% meaning that most glioblastoma cells were insensitive to the IL-2 activated allogeneic primary NK cells.

Supplementary figure 6 shows data for NZB19 glioblastoma cells highlighting differential sensitivity to primary NK cells obtained from two different blood donors. Donor A's NK cells were less effective at killing the glioblastoma cells compared to donor B. The violin plots also highlight the highly variable cell counts measured in different regions across the respective wells. This was due to clumping of the glioblastoma cells, which do not grow as nice even monolayers of cells. Each violin represents glioblastoma cell counts from 3 or 4 wells per treatment, with 9 images acquired per well (27-36 data points). The quantification of cell counts required this level of data acquisition to provide greater confidence as the glioblastoma cell loss was typically minimal at most of the ET ratios across most of the experiments. The automated pipeline also removed any user-bias in the image acquisition as the same regions were acquired across each of the wells.

The example images in supplementary figure 6C show the morphology of NZB19 cells in the media control-treated wells and those treated with the high ET ratio of 5:1. The images reveal how challenging it is to identify single glioblastoma cells based on their actin-cytoskeletal structure (green actin stain) as many of the glioblastoma cells clump together. However, the glioblastoma cells are much larger than the primary NK cells and therefore we were able to discriminate the glioblastoma cells based on the size of their nuclei, which was typically at least 15-20μm in diameter. Whereas the IL-2 activated NK cells have much smaller nuclei and they are typically less than 5μm in diameter. Supplementary figure 6C is also from the primary NK killing experiment where was saw the greatest glioblastoma cell loss, which averaged ~80% at 5:1 ratio at 24 hours-post addition of the activated NK cells. However, as shown in the images there were always residual glioblastoma cells that proved resistant to the NK cells.

Throughout the primary NK cell work we experienced considerable issues around the yield of purified NK cells from the blood PBMC preparations. Our ethical approvals limited the volume of blood drawn and thus the yield of PBMC. It also became evident that being able to conduct higher throughput analysis of NK killing of a greater range of glioblastoma cultures using the same NK donor would prove impossible. Additionally, even follow up experiments to repeat observations or understand the molecular basis to NK sensitivity or resistance would also prove challenging as blood collections were de-identified.
