# Supplementary material for: Trying to Kill a Killer; Impressive Killing of Patient Derived Glioblastoma Cultures Using NK-92 Natural Killer Cells Reveals Both Sensitive and Highly Resistant Glioblastoma Cells
Source: Cells. 2025 Jan 5;14(1):53. doi: 10.3390/cells14010053 (PMC11720543; doi:10.3390/cells14010053)
Supplement: Supplementary file 1 [file cells-14-00053-s001.zip › cells-3364126-supplementary.pdf]

Supplemental figures.

Trying to kill a Killer; impressive killing of patient derived glioblastoma cultures using NK-92 natural killer cells reveals both sensitive and highly resistant glioblastoma cells.

Authors- Jane Yu, Hyeon Joo Kim, Jordyn Reinecke, James Hucklesby, Tennille Read, Akshata Anchan, Catherine E Angel, **E Scott Graham**[#].

s.graham@auckland.ac.nz

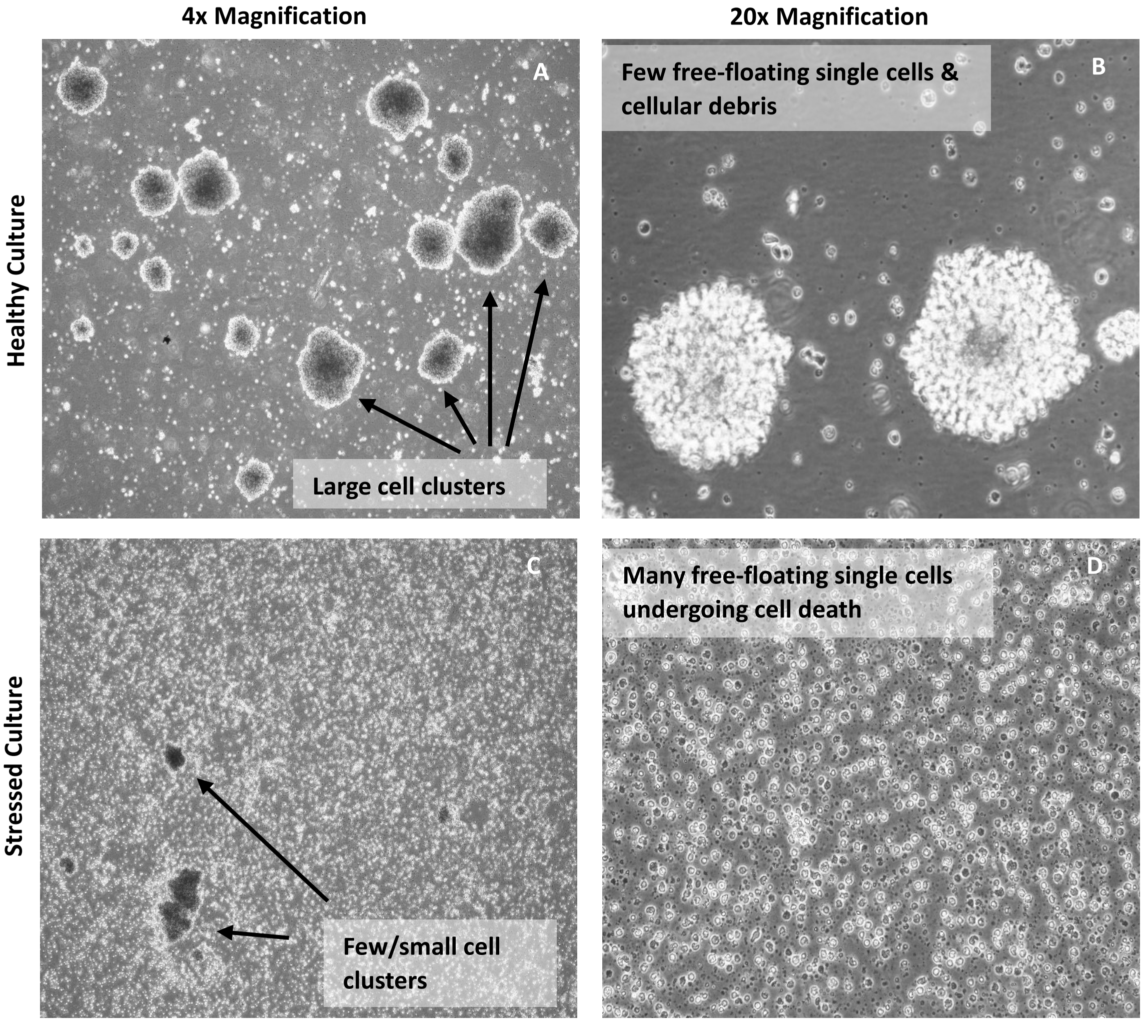

Supplementary Figure S1. Images of NK-92 suspension culture at various stages of growth imaged under phase microscope after 96 hours of culture. NK-92 cells cultured in RPMI based media with IL-2. Images A and C are taken at 4x magnification whereas images B and D are taken at 20x magnification. Images A and B on the top row are taken from the same suspension and represent a healthy NK-92 cells in a typical healthy culture. Where as, images C and D are taken from an overcrowded and stressed culture and represent unhealthy non-viable NK-92 cells.

Supplemental figure S2

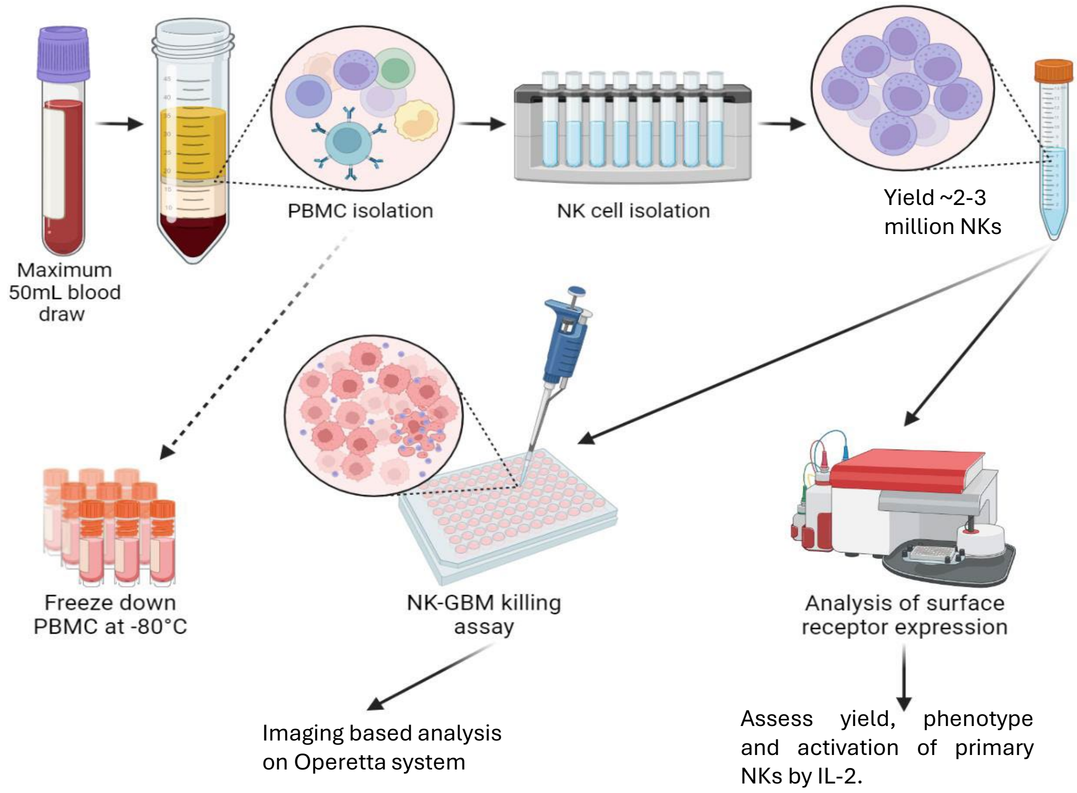

Supplementary Figure S 2. Schematic of primary NK cell isolation and usage. Yield of primary NK cells often a limiting factor for downstream applications. NK cells isolated from healthy donors' blood were required for initial killing assays however yield was also reduced due to assessment of harvest NK phenotype by flow cytometry. Typical blood yield of NKs from 40-50mls of blood was 2-3 million viable NKs, where 20-30% of this yield was required for phenotypic confirmation of NK purity, phenotype and activation.

Supplemental figure S3

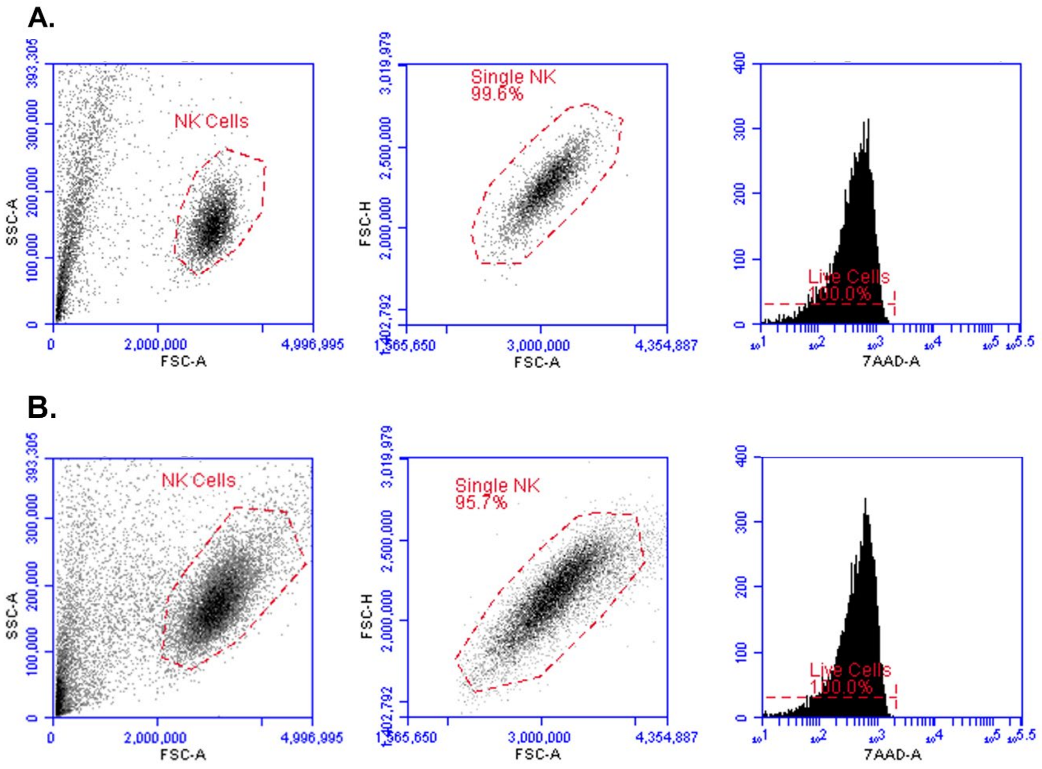

Supplementary Figure S3. Gating strategy of NK cells for flow cytometry analysis. The NK cell population was differentiated from debris by using the forward (FSC-A) vs. side (SSC-A) scatter. From the "NK Cells" gate, doublets were removed by using the forward scatter height (FSC-H) vs the area (FSC-A). From the resulting cell population in "Single NK", 7-AAD stained cells below the autofluorescence level was gated into the "Live Cells" population. This resulting population was used for all following flow cytometry analyses. Shown above is one representative replicate from donor 7. **A:** analysis of NK cells immediately after the untouched isolation protocol (non-activated NK cells). **B:** analysis of NK cells that were incubated with IL-2 for approximately 48h.

Supplemental figure S4

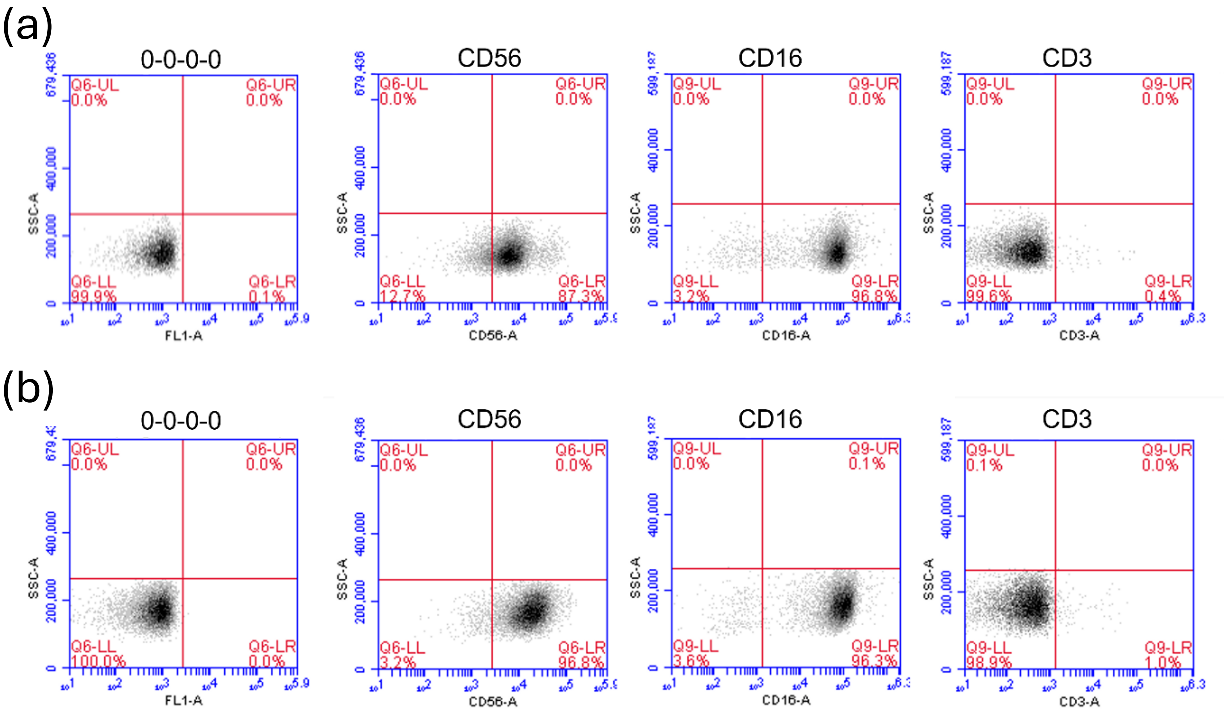

Supplementary Figure S 4. Primary NK cell purity analysis by flow cytometry. The bottom left quadrant indicates auto-fluorescent cells. The quadrant threshold was dictated by the autofluorescence from cells with no antibodies added (0-0-0-0). Cells stained for markers CD56 and CD16 that were above the autofluorescence level while remaining below the autofluorescence level for the T cell marker CD3 were deemed to be NK cells. Shown above is one representative replicate from donor 7. (a) analysis of NK cells immediately after the untouched isolation protocol (non-activated NK cells). (b) analysis of NK cells that were incubated with IL-2 for approximately 48h.

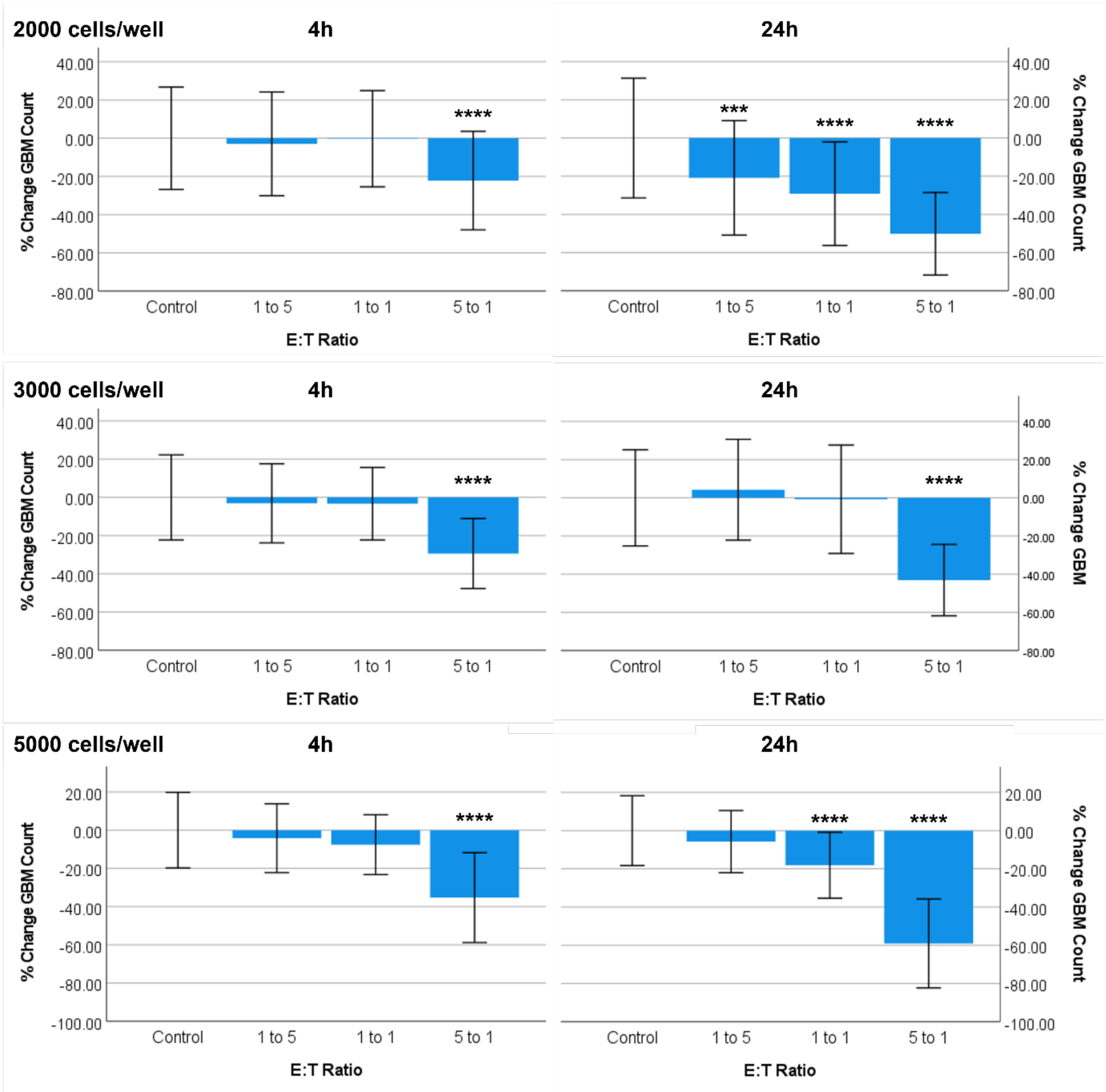

Supplementary Figure S 5. NK cell killing of NZB11 glioblastoma cells. Bar graph of mean percentage change in NZB11 glioblastoma cell counts compared to controls following a 4h and 24h killing assay. Results shown are compiled from 3 experimental repeats using NK cells from 3 different donors (donor 7, 8, and 9's NK cells). Error bars indicate ± SD. Cell counting was completed by CellProfiler using the imaging pipeline. An Independent-Samples Kruskal-Wallis test was performed, and pairwise mean comparisons were made. Statistically significant differences comparing experimental data and controls are indicated by asterisks. *P*-value = 0.05 (*), 0.01 (**), 0.001 (***), 0.0001 (****). No asterisks = no statistical significance.

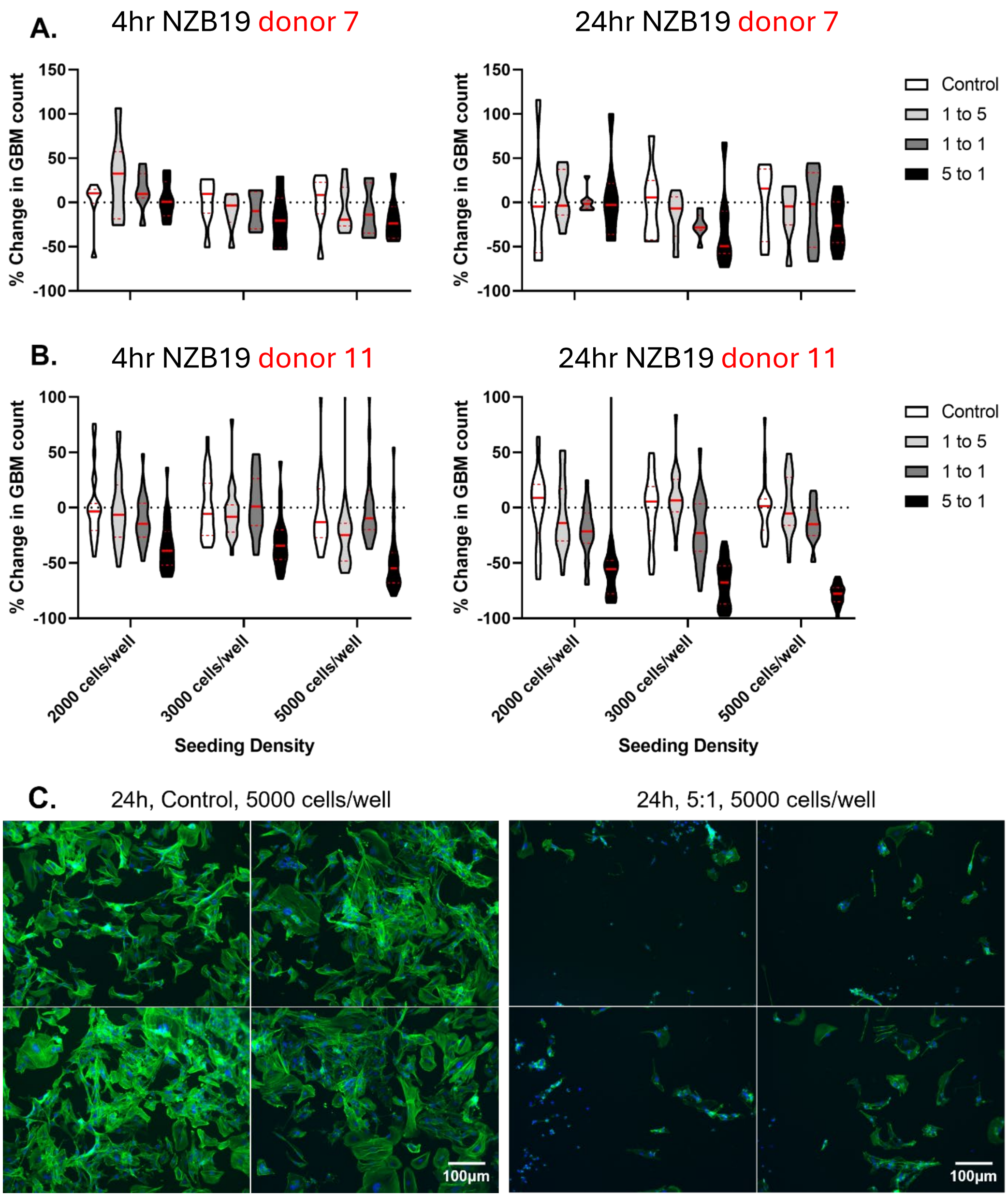

Supplementary Figure S6. Primary NK cell killing of NZB19 glioblastoma cells with different NK donors. Bar graph of mean percentage change in NZB11 glioblastoma cell counts compared to controls following a 4h and 24h killing assay. The data in (A) and (B) are from individual experiments showing the difference in cell loss from two different NK donors. In (A) the NK cells were from donor 7 and in (B) they were from donor 11. The violin plots show the variance in cell counts across the wells where each violin plot shows counts from 27-36 individual images. Cell counting was completed by CellProfiler using the imaging pipeline. The images in (C) show control glioblastoma cultures (green actin-stained cells; no NK cells) and those cells remaining after 24 hours incubation with NK cells from donor 11.

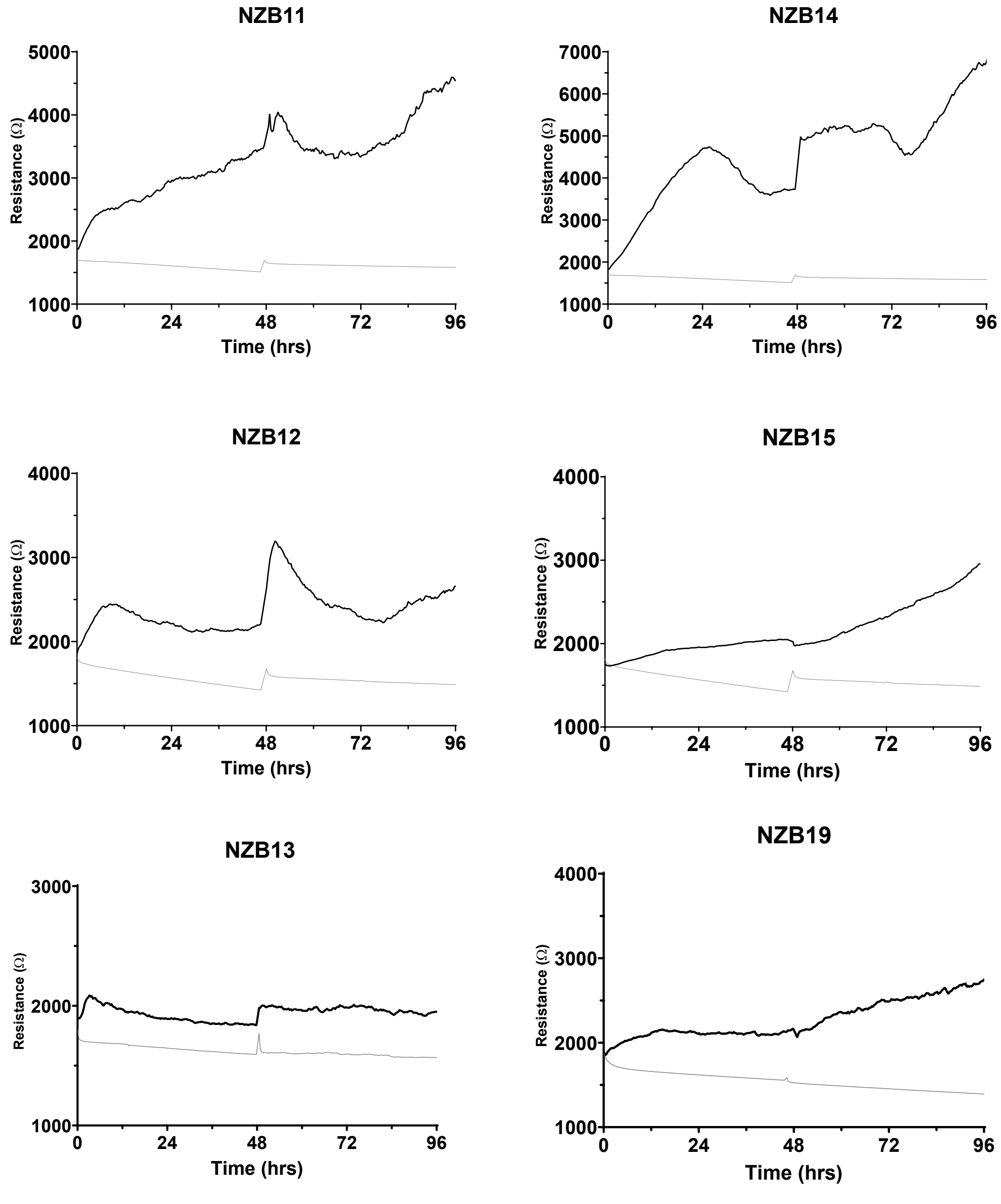

Supplementary Figure S7. ECIS cell adhesion curves for each glioblastoma culture. Each glioblastoma culture was seeded in 96 well ECIS 1E+ arrays and adhesion was monitored for 96 hours and represented as Resistance (Ω). The NZB11, NZB12, NZB14 and NZB15 cultures show the adhesion curves for seeding of 10,000 cells per well. Whereas the NZB13 and NZB19 show adhesion curves with 20,000 cells seeded per well as their degree of adhesion and influence on resistance was much less than the other cultures.
